# Bioactive Marine Drugs and Marine Biomaterials for Brain Diseases

**DOI:** 10.3390/md12052539

**Published:** 2014-05-02

**Authors:** Clara Grosso, Patrícia Valentão, Federico Ferreres, Paula B. Andrade

**Affiliations:** 1 REQUIMTE/Laboratory of Pharmacognosy, Department of Chemistry, Faculty of Pharmacy, University of Porto, Rua de Jorge Viterbo Ferreira, no. 228, 4050-313 Porto, Portugal; E-Mails: acfmtgrosso@ff.up.pt (C.G.); valentao@ff.up.pt (P.V.); 2 Research Group on Quality, Safety and Bioactivity of Plant Foods, Department of Food Science and Technology, CEBAS (CSIC), P.O. Box 164, Campus University Espinardo, Murcia 30100, Spain; E-Mail: federico@cebas.csic.es

**Keywords:** aragonite, conotoxins, neurodegeneration, neuroinflammation, Aβ peptide, tau hyperphosphorylation, protein kinases, receptors, voltage-dependent ion channels, cyclooxygenases

## Abstract

Marine invertebrates produce a plethora of bioactive compounds, which serve as inspiration for marine biotechnology, particularly in drug discovery programs and biomaterials development. This review aims to summarize the potential of drugs derived from marine invertebrates in the field of neuroscience. Therefore, some examples of neuroprotective drugs and neurotoxins will be discussed. Their role in neuroscience research and development of new therapies targeting the central nervous system will be addressed, with particular focus on neuroinflammation and neurodegeneration. In addition, the neuronal growth promoted by marine drugs, as well as the recent advances in neural tissue engineering, will be highlighted.

## 1. Introduction

Along with the increase of average life expectancy, the prevalence of neurological/neurodegenerative diseases is rising, prompting the recent research focused on developing novel drugs targeting the central nervous system (CNS) [1]. Inspired by the vastness and biodiversity richness of the marine environment, researchers have pursued the pharmacological potential of marine metabolites [2].

Pharmacological studies with marine compounds affecting the CNS involve areas of neuropharmacology, such as those of stimulation of neurogenesis, modulation of receptors and voltage-dependent ion channels and enzymes inhibition [3]. For instance, conotoxins peptides are currently being used as standard research tools in neuroscience, since they can interfere with receptors and channels, allowing a better understanding of how antagonist/agonist drugs bind to the binding sites [4]. These researches have already culminated with Food and Drug Administration (FDA) approval of Ziconitide (Prialt^®^), a synthetic equivalent of the ω-conotoxin MVIIA (isolated from *Conus magus* L.), for pain and stroke treatment [4,5]. Moreover, several other marine compounds are being evaluated in preclinical trials, such as the α-conotoxin Vc1.1 (isolated from *Conus victoriae* Reeve) and the χ-conotoxin MrIA/B (from *Conus marmoreus* L.), for the treatment of neuropathic pain, and the anti-epileptic conantokin-G, isolated from *Conus geographus* L. Currently undergoing a more advanced evaluation, *i.e.*, phase II trials, are ω-conotoxin CVID (from *Conus catus* Hwass in Bruguière) for neuropathic pain treatment, and contulakin-G (from *C. geographus*) for neuropathic and chronic inflammatory pain treatments [5], as well as 3-(2,4-dimethoxybenzylidene)-anabaseine (DMXBA), the synthetic derivative produced from the alkaloid anabaseine (isolated from nemertines), to treat schizophrenia [6] and Alzheimer’s disease (AD) [7].

This review covers the studies performed with marine invertebrate drugs from the year 2000 until the present, focusing on their role in fighting neuroinflammation states and neurodegeneration. One hundred and eighty-four examples of marine drugs affecting neuronal growth and synaptic functions, neuroinflammation, CNS enzymes and CNS voltage and ligand-gated ion channels will be given. Towards the conclusion of this paper, the usefulness of marine skeletons in neural tissue engineering will be discussed. Recently, some review papers have been published focusing on some of the aspects considered in this review. The modulation of receptors, voltage-dependent channels and enzymes by conopeptides is, by far, the most extensively reviewed subject [4,8,9,10]. Sakai and Swanson [11] presented a broad spectrum of marine drugs affecting those targets. Arias *et al.* [12] focused their attention on marine drugs affecting ion channels, and Al-Sabi *et al.* [13] reviewed data about marine toxins that target voltage-gated sodium channels. Kochanowska-Karamyan and Hamann [14] covered the role of marine indole alkaloids as potential new antidepressant and anti-anxiety drug leads. Bharate *et al.* [15] and Skropeta *et al.* [16] gathered information concerning sponge drugs with protein kinase inhibitory activity. A broader spectrum of enzyme inhibited by marine drugs was covered by Nakao and Fusetani [17]. Senthilkumar and Kim [18] compiled information concerning marine invertebrate natural drugs for inflammatory and chronic diseases, including AD. Finally, information regarding preclinical and clinical candidates in the field of neurology was published by Martínez [19], Twede *et al.* [10] and Bharate *et al.* [15].

## 2. The Nervous System

The nervous system is the network of specialized cells that conduct nerve impulses between parts of the body. The central nervous system (CNS) is responsible for driving and interpreting signals and for supplying excitatory stimuli to the peripheral nervous system (PNS); PNS nerves innervate muscle tissue, conducting sensory and excitatory stimuli to and from the spinal cord [20].

Besides neurons, whose function is to propagate nerve impulses, CNS and PNS also contain another type of cells called glial cells or neuroglia. Neuroglia comprises four types of cells, namely, astrocytes, oligodendrocytes, microglia cells in the CNS and Schwann cells in the PNS. Astrocytes are a very heterogeneous population of cells and they can interfere in axon guidance, synaptic support, control of the blood–brain barrier (BBB) and blood flow [21]. These are excitable cells like neurons, but they communicate by spontaneous or evoked cytosolic Ca^2+^ variations, instead of membrane electrical signals [22]. Oligodendrocytes and Schwann cells are responsible for the production of myelin [21,23]. Microglia cells are the immune cells of the CNS, contributing to CNS homeostasis during development, adulthood and ageing [24]. They protect the brain from damage and infection, by engulfing dead cells and debris. They are also implicated in synaptic remodelling during the development of the nervous system and they are activated in many neurodegenerative diseases [21,23]. In the nervous system, glial cells are more abundant than neurons and have some capacity for cell division. Conversely, neurons have no capacity for mitotic division, but can regenerate portions under certain conditions [20].

## 3. Regeneration of the CNS: Drawbacks and Challenges

Complete recovery from a CNS injury or neurological disorders has not yet been made possible [25]. This is because an injury is a continuous process, with a primary damage triggering a cascade of deleterious events, such as blood–brain barrier disruption, excitotoxicity, inflammation, oedema, ischemia, increase of free radicals and altered cell signalling and gene expression [26,27]. Therefore, a massive death of neuronal and glial cells may occur along with the loss of both the 3D spatial organization and the connectivity of neuronal networks [28].

Although neurite growth inhibitors are present in both CNS and PNS, the capacity for CNS nerves to regenerate is lower than that of peripheral nerves for several reasons. First, because astrocytes become “reactive astrocytes,” which produce glial scars that constitute a physical barrier to growth and up-regulate several extracellular-matrix-associated inhibitors of regeneration, such as chondroitin sulfate proteoglycans [29]. Second, conversely to a PNS injury, in the case of a CNS injury, BBB and blood–spine barrier function as constrainers to the recruitment of macrophages from the blood circulation to remove myelin and axonal debris and resident microglia can only give a delayed and slow response [24,30,31]. Moreover, in contrast to PNS, the up-regulation of regeneration-associated proteins (RAGs), which play a positive role in neurite outgrowth and axon regeneration, is relatively modest in the CNS after injury [32,33].

In order to counteract this low regenerating environment after a CNS injury, clinical trials have taken advantage of the recent progress in regenerative medicine, and new approaches for the treatment of CNS injuries have been explored, such as (i) cellular replacement with stem cells, (ii) delivery of brain-derived neurotrophic factor (BDNF), (iii) axon guidance with cell adhesion molecules and removal of growth inhibition molecules, (iv) manipulation of intracellular signalling with transcription factors, (v) bridging with a peripheral nerve bridge or foetal tissue or use of artificial substrates to guide axons across the scar, and (vi) modulation of the immune response [25,34]. Even though transplantation is a promising approach, therapeutic effects are currently limited due to the high level of donor cell death and lack of integration with the host brain tissue [27]. Conversely, PNS injuries are usually treated surgically by reconnection of the damaged nerve ends (78%) or by using an autograft (15%) or conduit (4%) [35,36,37]. Approximately 50% of surgical cases achieve normal to good function restoration [35].

## 4. Marine Drugs: Neuritogenic Activity, Neurotrophin-Mimic and Neurotrophin-Enhancer Agents

**Figure 1:**
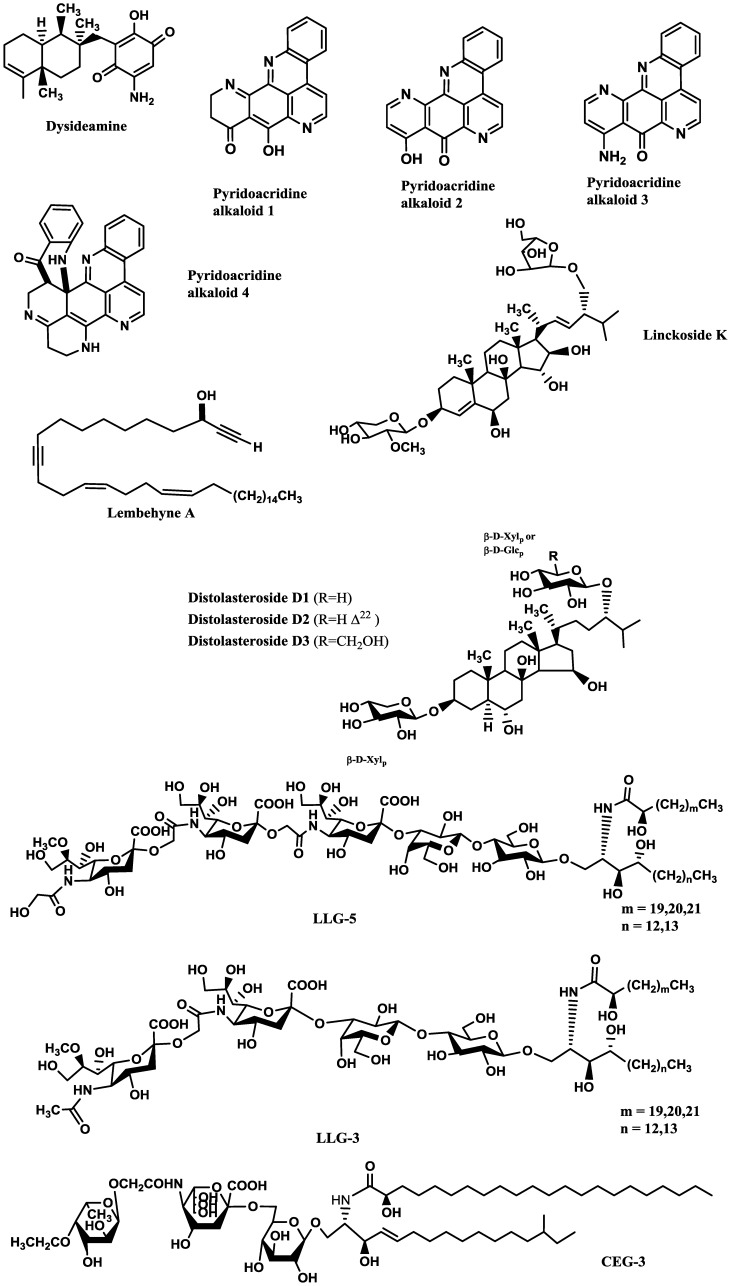
Potent marine drugs affecting neuronal growth and synaptic functions.

Compounds inducing neuronal growth are expected to become a new lead for medical treatment of CNS disorders, such as ischemic stroke and neurodegenerative diseases. Dysideamine (Figure 1), a sesquiterpene aminoquinone from the marine sponge *Dysidea* sp. 05C33, was shown to induce neurite outgrowth in mouse neuroblastoma Neuro 2A cells [38]. More than 40% of the cells treated with 3 μM of this compound presented neurite outgrowth but, at 10 μM, slight cytotoxic effects were observed [38]. Using the same cell system, as well as rat pheochromocytoma PC12 cells, Aoki *et al.* [39] studied the neuritogenic activity of four pyridoacridine alkaloids (Figure 1) isolated from the marine sponge *Biemna fortis* Topsent. None of these compounds were able to induce neurite outgrowth in rat pheochromocytoma PC12 cells. On the other hand, neurite outgrowth was induced in more than 50% of the Neuro 2A cells treated with compound 3 (0.01 μM), but at concentrations higher than 0.3 μM it was cytotoxic. Compounds 1 (labuanine A), 2 and 4 were less active. Taking into account the structure of these pyridoacridine alkaloids and the displayed activity, the authors suggested that the wide difference in neuritogenic activity between compounds 2 and 3 should be due to the presence of the amino group at C-9 in 3. Moreover, compound 3 provoked a four-fold increase of acetylcholinesterase (AChE) activity at 0.03 μM compared with the control, indicating that it induced both morphological and functional neuronal differentiation. Since neuronal differentiation closely relates to the cell cycle, the effect of the pyridoacridine alkaloids on the cell cycle of Neuro 2A cells was evaluated, revealing that, like topoisomerase II inhibitors, they arrested the cell cycle at G2/M phase. Thus, a possible mechanism suggested by the authors was that the induced neuronal differentiation could be related with inhibition of topoisomerase II.

In a similar study, lembehyne A (Figure 1), a linear polyacetylene isolated from the sponge *Haliclona* sp., induced neuritogenesis in both PC12 and Neuro 2A cell lines, at 2 and 0.1 µg/mL, respectively. Since treatment with an inhibitor of actin polymerization (cytochalasin B) or with an inhibitor of protein synthesis in eukaryotes (cycloheximide) inhibited the effect of lembehyne A, a mechanism dependent on actin polymerization and *de novo* protein synthesis was suggested for this compound [40,41]. Aditionally, lembehyne A (1 and 3 µg/mL) arrested the cell cycle at the G1 phase, a response also known to be induced by nerve growth factor (NGF), and induced a two- and four-fold increase of AChE activity at 1 and 3 µg/mL, respectively [41]. Later, the same research group investigated the structure–activity relationship among lembehynes A–C and five analogs using Neuro 2A cell system. They concluded that the features contributing to the activity were the carbon-chain length, since analogs with shorter carbon-chain were more active than lembehynes A–C, and that the presence of a hydroxyl group at C-3 was essential [42].

NGF and BDNF are essential for neuronal differentiation, growth, survival, function maintenance and prevention of ageing in the CNS and PNS [43,44].

Although NGF and BDNF are expected to have therapeutic potential in the treatment of neuronal injuries, they do not cross the BBB due to their size. Therefore, low molecular weight compounds mimicking their activity should be interesting as promising therapeutic agents to treat traumatic or ischemic brain injuries and neurodegenerative diseases [44]. In recent years, several low molecular weight substances from various natural sources have been shown to possess neurotrophic ability. Several marine drugs have proved to mimic and/or enhance NGF or BDNF activities.

Palyanova *et al.* [44] evaluated the neurotrophic potential of six sterols from *Asterina pectinifera* Muller and Troschel (starfish) using C1300-NB cell line. C1300-NB, in contrast to PC12 cells, have the capacity to spontaneously differentiate; a residual differentiation of 14%–25% was thus observed. This differentiation was increased by distolasterosides D_1_–D_3_ (>5 nM; Figure 1) more efficiently than by asterosaponin Р1 (>50 nM), (25*S*)-5α-cholestane-3β,4β,6α,7α,8,15α,16β,26-octaol (>10 nM), and (25*S*)-5α-cholestane-3β,6α,7α,8,15α,16β,26-heptaol (>50 nM). These compounds also synergistically enhanced NGF and BDNF activities.

Table 1 and Table 2 report the neurotrophin mimic and neurotrophin-enhancement effects of several marine drugs in PC12 cells [43,45,46,47,48,49,50].

**Table 1:** Marine drugs with neurotrophin mimic activity in PC12 cell line.

Compound/organism	Concentration tested (µM)	Neurites longer than soma diameter (%)
Linckoside A/blue starfish *Linckia laevigata* L.	40	25.0 [47]
Linckoside B/blue starfish *L. laevigata* L.	40	76.0 [47]
Linckoside F/blue starfish *L. laevigata* L.	40	30.0 [43]
Linckoside G/blue starfish *L. laevigata* L.	40	<10.0 [43]
Linckoside H/blue starfish *L. laevigata* L.	40	<10.0 [43]
Linckoside I/blue starfish *L. laevigata* L.	40	40.0 [43]
Linckoside J/blue starfish *L. laevigata* L.	40	<10.0 [43]
Linckoside K/blue starfish *L. laevigata* L.	40	50.0 [43]
NGF	10 *	45.0 [47]

* ng/mL.

**Table 2:** Synergistic effect between NGF and marine drugs in PC12 cells.

Compound/organism	[NGF] ng/mL	Effect of NGF alone (%)	[Drug] µM	Effect of NGF + marine drug (%)
Linckoside A/blue starfish *Linckia laevigata* L.	2.5	5.0	40	62.0 [47]
Linckoside B/blue starfish *L. laevigata* L.	2.5	5.0	40	87.0 [47]
Linckoside F/blue starfish *L. laevigata* L.	1.5	6.0	40	90.0 [43]
Linckoside G/blue starfish *L. laevigata* L.	1.5	6.0	40	40.0 [43]
Linckoside H/blue starfish *L. laevigata* L.	1.5	6.0	40	46.0 [43]
Linckoside I/blue starfish *L. laevigata* L.	1.5	6.0	40	95.0 [43]
Linckoside J/blue starfish *L. laevigata* L.	1.5	6.0	40	46.0 [43]
Linckoside K/blue starfish *L. laevigata* L.	1.5	6.0	40	98.0 [43]
LLG-5/blue starfish *L. laevigata* L.	5.0	20.6	10	59.3 [46]
LLG-3/blue starfish *L. laevigata* L.	5.0	20.6	10	63.1 [46]
Granulatoside A/blue starfish *L. laevigata* L.	1.5	<10.0	40	95.0 [45]
GP-3/starfish *A. pectinifera* Muller and Troschel	5.0	20.6	10	38.2 [48]
CEG-6/sea cucumber *Cucumaria echinata* Von Marenzeller	5.0	7.5	10	43.0 [49]
HLG-3/sea cucumber *C. echinata* Von Marenzeller	5.0	7.5	10	42.0 [49]
CEG-8/sea cucumber *C. echinata* Von Marenzeller	5.0	7.5	10	40.2 [49]
CEG-9/sea cucumber *C. echinata* Von Marenzeller	5.0	7.5	10	35.1 [49]
SJG-1/sea cucumber *C. echinata* Von Marenzeller	5.0	7.5	10	39.1 [50]
SJG-2/sea cucumber *Stichopus japonicus* Selenka	5.0	20.6	10	64.8 [50]
CG-1/sea cucumber *C. echinata* Von Marenzeller	5.0	7.5	10	43.0 [50]
CEG-3/sea cucumber *C. echinata* Von Marenzeller	5.0	7.5	10	50.8 [50]
CEG-4/sea cucumber *C. echinata* Von Marenzeller	5.0	7.5	10	34.0 [50]
CEG-5/sea cucumber *C. echinata* Von Marenzeller	5.0	7.5	10	35.7 [50]

Some of the studies allowed establishing structure-activity relationships. Han *et al.* [43] tested six steroid glycosides (Linckosides F–K) from the blue starfish *Linckia laevigata* L. and concluded that the carbon branch modified by a pentose at the side chain (present only in linckoside K; Figure 1) and the 2′-*O*-methyl ether group of xylose at C-3 (present in linckosides F and K) were the most important structures for the NGF-mimic activity. 2′-*O*-Methyl ether group of xylose at C-3 plays a role for the significant NGF-enhancing activity. Another steroid glycoside, granulatoside A [45], and two gangliosides, LLG-3 (Figure 1) and LLG-5 (Figure 1) [46], isolated from the same blue starfish, were also very promising.

Kisa *et al.* [50] evaluated the NGF-mimic activity of five monosialo-gangliosides from the sea cucumber *Cucumaria echinata* Von Marenzeller, SJG-1, CG-1, CEG-3, CEG-4 and CEG-5. The most active one was CEG-3 (Figure 1), which possesses an acetyl group at the terminal fucose unit. Among the disialogangliosides (HLG-3 and CEG-6) and trisialogangliosides (CEG-8 and CEG-9) isolated from the same sea cucumber [49], those displaying highest activity were CEG-6, HLG-3 and CEG-8, although lower than that of CEG-3. This was in accordance with the previous assumption made by the same authors, since CEG-6 and HLG-3 possess a terminal fucose without acetyl group and CEG-8 does not contain a terminal fucose. Despite their structural similarity, the different NGF-enhancement effect of linckosides A and B suggests that the sugar moiety at C-29 of the aglycon plays an important role for the activity of these steroid glycosides [47].

## 5. Marine Drugs Affecting Enzymes Involved in Neurodegeneration

Neurodegenerative diseases, such as AD and Parkinson’s disease (PD), are characterized by the loss of particular neuronal populations and by intraneuronal and extracellular accumulation of fibrillary materials [51]. AD is the most common form of dementia. It is an age-related neurodegenerative disorder characterized by extracellular deposition of plaques of aggregated β-amyloid protein (Aβ), intracellular deposition of neurofibrillary tangles that contain hyperphosphorylated tau (τ) protein, and a profound loss of basal forebrain cholinergic neurons that innervate the hippocampus and the neocortex [52]. Current AD treatment consists of the administration of inhibitors of AChE and butyrylcholinesterase (BuChE) enzymes in order to counteract brain’s acetylcholine deficiency [53]. However, other enzymes could be considered a target for future drug development, such as the proteases β-secretase (BACE1) and presenilin-dependent γ-secretase [54,55,56] involved in the cleavage of amyloid-β precursor protein (APP) into Aβ fragments, and protein kinases that hyperphosphorylate τ protein making up paired helical filaments (PHFs) and straight filaments of neurofibrillary tangles (NFTs) in the brain [57,58,59].

Protein kinases also display a pivotal role in other neurodegenerative disorders, such as in PD. Hyperphosphorylated α-synuclein, the major constituent of Lewy bodies, is one of the most important hallmarks of PD [60,61]. Several post-translational modifications to α-synuclein occur in PD, phosphorylation at serine (Ser)-129 residue being among them [61,62].

In the next sections, examples of marine compounds with inhibitory activity against cholinesterases (AChE and BuChE), BACE1 and protein kinases will be given.

### 5.1. Inhibition of Cholinesterases (ChEs) Activity

Beedessee *et al.* [53] evaluated the anticholinesterase effect of 134 extracts from 45 species of marine sponges and two of them showed strong AChE inhibition, namely the ethyl acetate extracts of *Pericharax heteroraphis* Poléjaeff (90% inhibition at 0.1 mg/mL) and of *Amphimedon navalis* Pulitzer-Finali (96% inhibition at 0.1 mg/mL). These extracts were rich in terpenoid compounds. Two other extracts obtained from the sponges *Latrunculia lendenfeldi* Hentschel and *Latrunculia bocagei* Ridley and Dendy displayed IC_50_ = 1.3 and 9 ng/mL, respectively [63].

**Figure 2:**
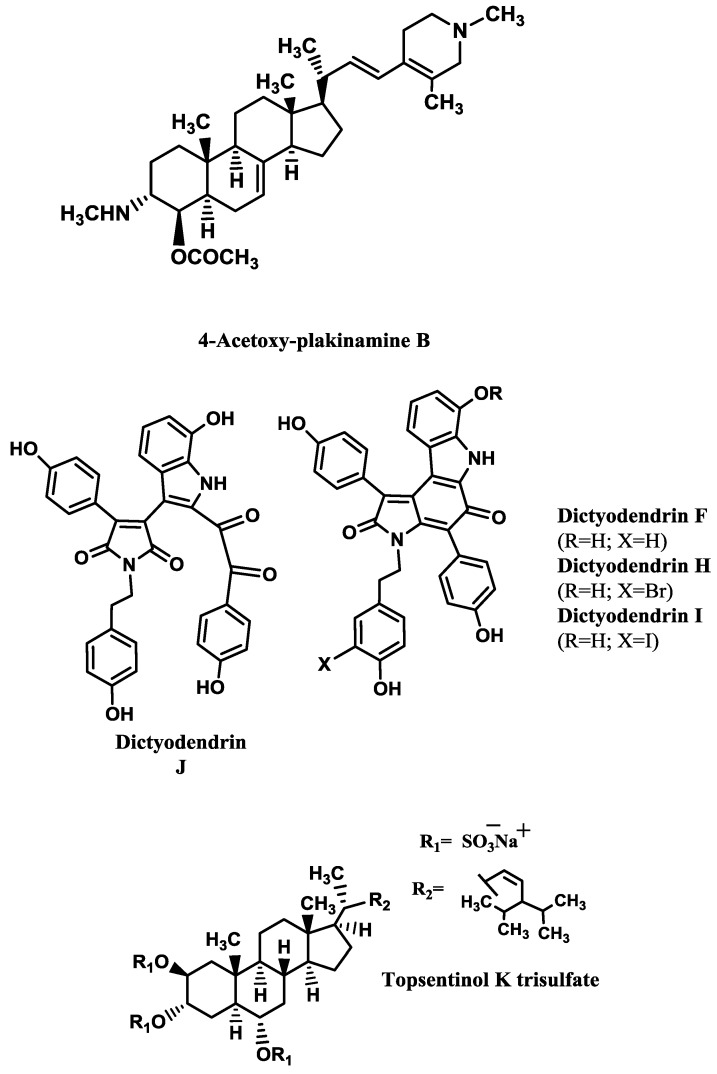
AChE and BACE1 inhibitors isolated from marine invertebrates.

Some examples of AChE inhibitors isolated from sponges, corals and molluscs are shown in Table 3. The kinetics analysis of AChE inhibition promoted by the stigmastane-type steroidal alkaloid 4-acetoxy-plakinamine B (Figure 2) suggested a mixed-competitive mode of inhibition [64].

**Table 3:** Marine drugs as AChE inhibitors.

Compound/Organism	IC_50_ (µM)
4-acetoxy-plakinamine B/sponge *Corticium* sp.	3.75 [64]
2-Bromoamphimedine/sponge *Petrosia n.* sp.	300 [65]
Petrosamine/sponge *Petrosia n.* sp.	91 [65]
Cladidiol/soft coral *Cladiella* sp.	67 [66]
Turbotoxin A/mollusc *Turbo marmoratus* L.	28 [67]

### 5.2. Inhibition of BACE1

Williams *et al.* [68] screened 130 pre-fractionated extracts from marine invertebrates and cyanobacteria against BACE1 activity, resulting in 7% of the extracts with outstanding inhibition (>90%) and 11% with activity between 70% and 89%. One group of submicromolar BACE1 inhibitors revealed by this study was the bastadins, a family of highly modified tetrapeptides occurring in some species of sponges, from which bastadin 9 is an example. Several metabolites isolated from sponges [69,70,71,72,73,74] showed BACE1 inhibitory activity (Table 4). The most promising ones are dictyodendrins F and H–J (Figure 2) [74] and topsentinol K trisulfate (Figure 2) [70].

Dai *et al.* [69] tested several xestosaprols and concluded that the β-orientation of the C-3 alcohol (only present in xestosaprol H) was an important feature for the activity. Structure-activity relationships were also established for topsentinols. Topsentinol K trisulfate was the only active sterol isolated from the sponge *Topsentia* sp., while topsentinols K and L were inactive. These results demonstrated that the presence of sulfate esters contribute to BACE1 activity [70].

**Table 4:** BACE1 inhibitors.

Compound/organism	IC_50_ (μM)
Xestosaprol D/sponge *Xestospongia* sp.	93.2 [72]
Xestosaprol F/sponge *Xestospongia* sp.	135.0 [69]
Xestosaprol G/sponge *Xestospongia* sp.	155.0 [69]
Xestosaprol H/sponge *Xestospongia* sp.	82.0 [69]
Xestosaprol I/sponge *Xestospongia* sp.	163.0 [69]
Xestosaprol J/sponge *Xestospongia* sp.	90.0 [69]
Xestosaprol K/sponge *Xestospongia* sp.	93.0 [69]
Xestosaprol L/sponge *Xestospongia* sp.	98.0 [69]
Xestosaprol M/sponge *Xestospongia* sp.	104.0 [69]
Dictyodendrin F/sponge *Ianthella* sp.	1.5 [74]
Dictyodendrin H/sponge *Ianthella* sp.	1.0 [74]
Dictyodendrin I/sponge *Ianthella* sp.	2.0 [74]
Dictyodendrin J/sponge *Ianthella* sp.	2.0 [74]
Dictazole A/sponge *Smenospongia cerebriformis* Duchassaing and Michelotti	135.0 [71]
Topsentinol K trisulfate/sponge *Topsentia* sp.	1.2 [70]
Lamellarin O/sponge *Ianthella* sp.	40% (at 10 μM) [73]
Lamellarin O1/sponge *Ianthella* sp.	60% (at 10 μM) [73]
Lamellarin O2/sponge *Ianthella* sp.	40% (at 10 μM) [73]
Ianthellidone F/sponge *Ianthella* sp.	40% (at 10 μM) [73]

### 5.3. Inhibition of Protein Kinases

The human kinome codifies nearly 500 different protein kinases, which have serine/threonine (Ser/Thr) or tyrosine (Tyr) specificity. They catalyse phosphorylation pathways that regulate most of the biological processes, but abnormal phosphorylation is, normally, a cause or a consequence of disease [61]. As stated above, inhibitors of these protein kinases can be useful to alleviate the symptoms of neurodegenerative disorders, such as AD and PD. In the next sections, a brief description on the involvement of protein kinases in neurodegeneration will be given, as well as some examples of marine protein kinases inhibitors and, when available, data about their inhibition mode.

#### 5.3.1. Glycogen Synthase Kinase 3 (GSK-3)

GSK-3, also known as τ phosphorylating kinase I, is a multifunctional Ser/Thr kinase that is involved in glycogen metabolism, insulin signalling, cell proliferation, neuronal function, oncogenesis and embryonic development. There are two isoforms (α and β) with 98% homology and similar biological functions, but most of the research has been dedicated to the isoform β. GSK-3 is highly expressed in the brain and is associated with several CNS disorders, such as AD, bipolar disorder, Huntington’s disease and other neurodegenerative diseases [75,76].

GSK-3β phosphorylates transcription factors and cytoskeletal proteins, such as τ [77]. There are, at least *in vitro*, 40 different Ser and Thr residues in τ that can be phosphorylated by GSK-3 [78,79,80,81].

The human τ gene suffers extensive alternative splicing, giving rise to the expression of multiple spliced exons, exon 10 being one of them. The presence of exon 10 results in τ with four repeat microtubule-binding sequences (4R), while isoforms without exon 10 have only three (3R). Normally, the ratio of 3R and 4R tau transcripts is close to one. Although mutations in splicing regulatory elements are common in inherited tauopathies, in sporadic AD the ratio 4R/3R is also increased [82]. In addition to hyperphosphorylate τ, GSK-3 can also induce τ splicing, because it phosphorylates the splicing factor SC35, an enhancer of splicing elements that regulate exon 10 splicing in τ [79]. Hernández *et al.* [83] demonstrated that GSK-3 inhibition in cultured neurons affected τ splicing, resulting in increased τ mRNA containing exon 10.

Moreover, GSK-3β has been reported to play a role in the toxic effect mediated by Aβ since, in cultured cells, Aβ activates GSK-3, leading to the phosphorylation of SC35 [79] and exposure of cortical and hippocampal primary neuronal cultures to Aβ induces activation of GSK-3β, τ hyperphosphorylation and cell death [78]. Thus, inhibition of GSK-3 can contribute to the reduced formation of both Aβ plaques and neurofibrillary tangles [84].

**Figure 3:**
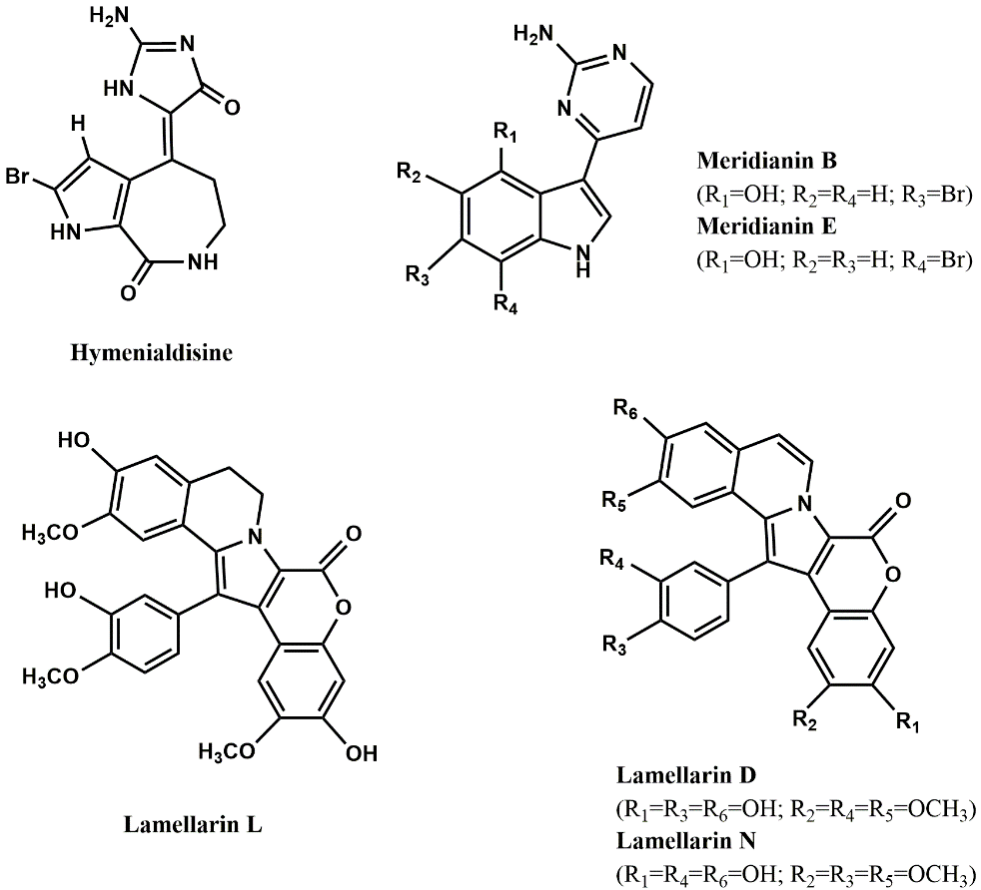
Most potent protein kinases inhibitors from marine organisms.

Marine compounds [76,78,80,81,85,86,87,88,89,90] able to inhibit both isoforms of GSK-3 are shown in Table 5 and Figure 3. As it can be seen, hymenialdisine (Figure 3), lamellarins (Figure 3) and meridianins (Figure 3) are the most active ones.

Few studies explored the mode of inhibition and the structural features contributing to high inhibitory activity of GSK-3 inhibitors. Concerning the first aspect, it is known that the alkaloid hymenialdisine and meridianins are competitive inhibitors at the ATP-binding site [81,91], while the alkaloid manzamine A [80] and the furanoterpenoids tricantin [89] and palinurin [76] are non-ATP competitive. According to Eldar-Finkelman and Martinez [91], ATP non-competitive GSK-3 inhibitors should be more selective than ATP-competitive ones, since they bind to unique regions within GSK-3, leading to a more subtle modulation of the kinase activity than by simply ATP entrance blockade.

**Table 5:** GSK-3 inhibitors from marine organisms.

Compound/organism	Isoform	IC_50_ (μM)
Carteriosulfonic acid A/sponge *Carteriospongia* sp.	GSK-3β	12.5 [88]
Carteriosulfonic acid B/sponge *Carteriospongia* sp.	GSK-3β	6.8 [88]
Carteriosulfonic acid C/sponge *Carteriospongia* sp.	GSK-3β	6.8 [88]
Hymenialdisine/sponge *Axinella verrucosa* Esper	GSK-3β	10.0* [81]
Tricantin/sponge *Ircinia* sp.	GSK-3β	7.5 [89]
Lamellarin α/ascidian *Didemnum obscurum* F. Monniot	GSK-3α/β	1.4 [86]
Lamellarin D/prosobranch mollusc *Lamellaria* sp.	GSK-3α/β	0.3 [86]
Lamellarin H/ascidian *Didemnum chartaceum* Sluiter	GSK-3α/β	9.5 [86]
Lamellarin L/ascidian *Didemnum* sp.	GSK-3α/β	40.0 * [86]
Lamellarin N/ascidian *Didemnum* sp.	GSK-3α/β	5.0 * [86]
Leucettamine B/sponge *Leucetta microraphis* Haeckel	GSK-3α	7.7 [85]
Leucettamine B/sponge *L. microraphis* Haeckel	GSK-3β	>10.0 [85]
Leucettamine B/sponge *L. microraphis* Haeckel	GSK-3α/β	2.9 [85], 15.0 [90]
Manzamine A/sponge *Acanthostrongylophora* sp.	GSK-3β	10.2 [80], 12.30 [78]
Meridianin A/ascidian *Aplidium meridianum* Sluiter	GSK-3β	1.3 [87]
Meridianin B/ascidian *A. meridianum* Sluiter	GSK-3β	0.5 [87]
Meridianin C/ascidian *A. meridianum* Sluiter	GSK-3β	2.0 [87]
Meridianin D/ascidian *A. meridianum* Sluiter	GSK-3β	2.5 [87]
Meridianin E/ascidian *A. meridianum* Sluiter	GSK-3β	2.5 [87]
Meridianin F/ascidian *A. meridianum* Sluiter	GSK-3β	2.0 [87]
Meridianin G/ascidian *A. meridianum* Sluiter	GSK-3β	350.0 [87]
Palinurin/sponge *Ircinia dendroides* Schmidt	GSK-3β	2.6 [76]
(*Z*)-5-(4-Hydroxybenzylidene)-hydantoin/sponge *Hemimycale arabica* Ilan, Gugel and van Soest	GSK-3β	13.70 [78]

* nM.

Regarding the second issue, Hamann *et al.* [80] synthetized several manzamine A analogs to study the influence of several substituents on GSK-3 inhibition. They concluded that the entire molecule (carboline moiety and aliphatic heterocyclic system) contributed for the inhibitory activity. Concerning the carboline moiety, the substitution of nitrogen 9 by large groups, such as isobutyl, dodecyl or methylcarboxybutyl, produced non-active compounds, while shorter groups (methyl and ethyl) did not cause activity reduction. Changes in the aliphatic heterocyclic system also influence GSK-3 inhibition, because if conformational restriction is increased, compounds are more active.

Baunbæk *et al.* [86] evaluated the ability of several lamellarins (Figure 3) and their analogs to inhibit not only GSK-3, but also other kinases (see next sections). Structure-activity studies led them to conclude that complex, but specific, interactions between lamellarins’ substituents and their kinase targets may exist, since different substituents influenced the inhibitory activity against different kinases.

Other protein kinases function as activators for τ phosphorylations by GSK-3, such as casein kinase 1 (CK1) e 2 (CK2), dual specificity tyrosine phosphorylation-regulated kinase 1 A (DYRK1A), AMP-dependent protein kinase (PKA) and cyclin-dependent kinase-5 (CDK5) [61,79]. For instance, when CDK-5 phosphorylates τ at Ser-235 and Ser-404 residues, it promotes the subsequent τ phosphorylation by GSK-3 at Thr-231 and Ser-400, respectively. On the other hand, if PKA phosphorylates τ at Ser-214, it will activate τ phosphorylation by GSK-3 at Ser-210, Thr-205, Ser-199 and Ser-195 residues. However, some τ residues, such as Ser-396 and Ser-404, can be directly phosphorylated by GSK-3 without prior activity of other kinases [77,79].

#### 5.3.2. DYRK1A

DIRK1A is located in chromosome 21 and codifies a protein kinase responsible for the phosphorylation of τ at Thr-212, Ser-202 and Ser-404 residues *in vitro* and *in vivo*. Studies indicate that overexpression of DYRK1A in the brains of Down’s syndrome patients may contribute to early onset of AD pathology through hyperphosphorylation of τ [59].

Moreover, DYRK1A also phosphorylates other AD-related proteins, *in vitro* and *in vivo*. Phosphorylation of APP at Thr-668 residue leads to APP cleavage by BACE1 and γ-secretase and consequently to increased production of Aβ peptide [92]. In a similar way, phosphorylation at Thr-354 residue of presenilin 1 (PS1), a key component of the γ-secretase complex, also induced an increased γ-secretase activity [93]. Phosphorylation of septin-4 (SEPT-4) at Ser-68 and Ser-107 residues by DYRK1A may regulate specific protein–protein interactions, since septins are a family of filament-forming guanine nucleotide-binding proteins involved in cytokinesis, exocytosis and other cellular processes, such as synapse functions. It was shown that a complex formed by SEPT4, DYRK1A and α-synuclein may contribute to the development of α-synuclein-positive cytoplasmic aggregates characteristic of PD and, since SEPT4 has been found in neurofibrillary tangles, SEPT4/DIRK1A is also involved in the pathology of AD [94,95,96]. Finally, DIRK1A also phosphorylates the regulator of calcineurin 1 (RCAN) at Ser-112 and Thr-192 residues, the latter enhancing τ phosphorylation [97] and phosphorylating Munc18–1 at Thr-479 residue, stimulating its binding to Syntaxin 1 and X11α, two proteins involved in synaptic vesicle exocytosis and APP processing, respectively [98]. Examples of marine compounds [85,86] that inhibit DYRK1A are shown in Table 6 and Figure 3.

**Table 6:** DYRK1A inhibitors from marine organisms.

Compound/organism	IC_50_ (μM)
Lamellarin α/ascidian *Didemnum obscurum* F. Monniot	5.0 [86]
Lamellarin D/prosobranch mollusc *Lamellaria* sp.	0.5 [86]
Leucettamine B/sponge *Leucetta microraphis* Haeckel	0.6–1.0 [85]
Lamellarin L/ascidian *Didemnum* sp.	0.1 [86]
Lamellarin N/ascidian *Didemnum* sp.	40.0 * [86]

* nM.

#### 5.3.3. CK1 and CK2

In mammals, the CK1 family of protein kinases consist of monomeric enzymes assembled from seven isoforms (α, β, γ1, γ2, γ3, δ, and ε). They are responsible for the phosphorylation of cytoskeletal proteins, such as spectrin, troponin, myosin, ankyrin, τ and α-synuclein, but also of non-cytoskeletal proteins (SV40 T antigen, p53, and β-catenin). These phosphorylations modulate important physiological functions like vesicular trafficking, DNA repair, cell cycle kinetics and cell division [99].

In AD patients’ brains, CK1α and CK1δ are co-localized with neurofibrillary lesions and granulovacuolar degeneration bodies. Furthermore, CK1α, CK1ε and CK1δ levels are increased in CA1 region of hippocampus, with a predominance of CK1δ. This CK1δ isoform phosphorylates τ at Ser-202, Thr-205, Ser-396 and Ser-404 residues and a combination of CK1δ and GSK-3 activities induce more than three-quarters of the Ser/Thr phosphorylations identified in τ-PHF, indicating that both protein kinases are involved in the pathogenesis of AD [61]. Additionally, APP, BACE1 and γ-secretase contain multiple CK1 phosphorylation sites and CK1ε leads to an increase of Aβ peptide production. On the other hand, Aβ stimulates CK1 activity [79,100].

CK1 is also involved in PD pathology. It has been demonstrated that α-synuclein is phosphorylated at Ser-129 by CK1 [61].

The CK2 holoenzyme forms a heterotetrameric complex with two catalytic (CK2α and CK2α′) and two regulatory (CK2β) subunits. Overexpression of CK2 leads to several pathological conditions, ranging from cardiovascular pathologies and cancer progression to infectious diseases and neurodegeneration. CK2 activity increases due to the presence of Aβ peptide and, thus, may accelerate τ phosphorylation. Besides CK2’s role in AD progression, CK2β subunits are present in Lewy bodies and phosphorylate α-synuclein at Ser-129 residue [61].

Table 7 and Figure 3 report some examples of marine compounds [81,86,87] that display inhibitory activity against CK1 and CK2.

Hymenialdisine is a competitive inhibitor at the ATP-binding site [81].

#### 5.3.4. Cyclin-Dependent Kinase 5 (CDK5)

CDKs are a group of protein kinases that regulate cell-cycle control (CDK1–4, 6 and 7), thymocyte apoptosis (CDK2), neuronal functions (CDK5) and transcriptional control (CDK7–9). CDK5, initially known as brain proline-directed protein kinase or neuronal cdc2-like protein kinase, has been considered a major τ kinase that contributes to tauopathies. Interaction of CDK5 with either p35 or p39, two activator proteins, is necessary for its activation [101]. CDK5/p35 is involved in several processes critical to CNS function during development and throughout maturity [102]. CDK5/p35 is known to phosphorylate τ (at Ser-235, Ser-396 and Ser-404) and MAP-1B, Pak1 kinase and neurofilament subunits [81] and its activity is promoted by Aβ peptide. Indeed, CDK5/p35 phosphorylates τ at Ser-396 and Ser-404 residues in response to Aβ25–35 [103].

**Table 7:** CK1 and CK2 inhibitors from marine organisms.

Compound/organism	Enzyme	IC_50_ (μM)
Hymenialdisine/sponge *Axinella verrucosa* Esper	CK1	35.0 * [81]
Hymenialdisine/sponge *A. verrucosa* Esper	CK2	7.0 [81]
Lamellarin α/ascidian *Didemnum obscurum* F. Monniot	CK1	7.9 [86]
Lamellarin D/prosobranch mollusc *Lamellaria* sp.	CK1	13.0 [86]
Lamellarin K/ascidian *Didemnum* sp.	CK1	6.0 [86]
Lamellarin H/ascidian *Didemnum chartaceum* Sluiter	CK1	5.3 [86]
Meridianin B/ascidian *Aplidium meridianum* Sluiter	CK1	1.0 [87]
Meridianin C/ascidian *A. meridianum* Sluiter	CK1	30.0 [87]
Meridianin D/ascidian *A. meridianum* Sluiter	CK1	100.0 [87]
Meridianin E/ascidian *A. meridianum* Sluiter	CK1	0.4 [87]

* nM.

Aberrant CDK5 activity is induced by the conversion of p35 to p25 by calpain, a Ca^2+^-dependent cysteine protease. CDK5/p25 plays a role in the pathogenesis of neurodegenerative diseases since it induces the formation of τ-PHF, τ aggregation and neuronal loss [102,104]. Other evidence from the involvement of Aβ peptide in τ hyperphosphorylation comes from the ability of Aβ to directly promote an increase of the levels of intracellular Ca^2+^ ([Ca^2+^]i) in neurons, this increment leading to calpain activation, which, in turn, cleaves p35 into p25 [105].

Table 8 and Figure 3 show some examples of CDK5 inhibitors isolated from marine organisms [80,86,87,106].

**Table 8:** CDK5 inhibitors from marine organisms.

Compound/organism	Enzyme	IC_50_ (μM)
Lamellarin α/ascidian *Didemnum obscurum* F. Monniot	CDK5/p25	>10.0 [86]
Lamellarin D/prosobranch mollusc *Lamellaria* sp.	CDK5/p25	0.6 [86]
Lamellarin L/ascidian *Didemnum* sp.	CDK5/p25	0.1 [86]
Lamellarin N/ascidian *Didemnum* sp.	CDK5/p25	25.0 * [86]
Fascaplysin/sponge *Fascaplysinopsis* sp.	CDK5/p35	20.0 [106]
Manzamine A/sponge *Acanthostrongylophora* sp.	CDK5/p35	1.5 [80]
Meridianin A/ascidian *Aplidium meridianum* Sluiter	CDK5/p25	3.0 [87]
Meridianin B/ascidian *A. meridianum* Sluiter	CDK5/p25	1.0 [87]
Meridianin C/ascidian *A. meridianum* Sluiter	CDK5/p25	6.0 [87]
Meridianin D/ascidian *A. meridianum* Sluiter	CDK5/p25	5.5 [87]
Meridianin E/ascidian *A. meridianum* Sluiter	CDK5/p25	0.2 [87]
Meridianin F/ascidian *A. meridianum* Sluiter	CDK5/p25	20.0 [87]
Meridianin G/ascidian *A. meridianum* Sluiter	CDK5/p25	140.0 [87]

* nM.

#### 5.3.5. PKA Inhibitors

PKA is the first element of cAMP signal transduction cascade, one of the several second messenger-dependent pathways that generate intracellular responses to extracellular signals. PKA mediates most of cAMP actions by phosphorylation [107].

Phosphorylation of τ at Ser-214 residue by PKA affects the interaction between τ and microtubules by reducing the tau’s affinity for them. This phenomenon also occurs with the phosphorylation caused by GSK-3β and CDK5 [108].

Examples of marine PKA inhibitors [87] are shown in Table 9 and Figure 3.

**Table 9:** PKA inhibitors from marine organisms.

Compound/organism	IC_50_ (μM)
Meridianin A/ascidian *Aplidium meridianum* Sluiter	11.0 [87]
Meridianin B/ascidian *A. meridianum* Sluiter	0.2 [87]
Meridianin C/ascidian *A. meridianum* Sluiter	0.7 [87]
Meridianin D/ascidian *A. meridianum* Sluiter	1.0 [87]
Meridianin E/ascidian *A. meridianum* Sluiter	90.0 * [87]
Meridianin F/ascidian *A. meridianum* Sluiter	3.2 [87]
Meridianin G/ascidian *A. meridianum* Sluiter	120.0 [87]

* nM.

## 6. Marine Drugs Modulating CNS Voltage-Dependent Ion Channels and CNS Receptors

Voltage-dependent ion channels are intrinsic membrane proteins that play a pivotal role in fast communication in excitable cells. The pore region determines cation selectivity and is the binding site for many channel blockers. Toxins that interact with the pore can be used to understand its spatial organisation and may also be useful to design drugs that modify the function of ion channels in pathological conditions, such as stroke, pain, or epilepsy [109]. Calcium, sodium and potassium channels are voltage-dependent ion channels.

### 6.1. Calcium Channels

At least four distinct types of high-voltage-activated Ca^2+^ channels (L-, N-, P/Q- and R-type) are expressed in cultured hippocampal neurons and are sensitive to different blockers, such as ω-conotoxin GVIA (N-type Cav2.2 channels), spider ω-Aga-IVA (P/Q-type Cav2.1 channels) and nimodipine (L-type Cav1.1–1.4 channels) [110,111]. Several types may contribute to neurotransmitter release, mainly P/Q- and R-type. Selective modulators may, therefore, allow the selective treatment of conditions, such as pain and stroke [109].

Many of the ischemia-induced pathophysiologic cascades that destroy the CA1 pyramidal neurons in hippocampus are triggered by pre- and post-synaptic Ca^2+^ influx. Therefore, many Ca^2+^ channels blockers, such as ω-conotoxins, have been shown to be neuroprotective in global models of ischemia [112,113].

Favreau *et al.* [114] injected ω-conotoxin CNVIIA intracerebroventricularly to mice, which caused shaking activity. At 1.5 pmol/g, the toxin produced mild tremors in mice that became more intense as the amount injected increased. This behaviour is characteristic of ω-conotoxins blockers of N-type voltage-sensitive Ca^2+^ channels and, consequently, the authors tested the selectivity of CNVIIA for different subtypes of Ca^2+^ channels. Binding of ^125^I-ω-Ctx CNVIIA to rat brain synaptosome indicated its reversibility. Moreover, CNVIIA exhibited a clear selectivity for N-type voltage-sensitive Ca^2+^ channels *vs.* P/Q-type, since it displaced ^125^I-ω-CNVIIA and ^125^I-ω-GVIA with the same affinity, but was not so efficient at inhibiting ^125^I- ω-MVIIC binding. Similarly, the ω-conotoxin SO-3 inhibited high-voltage-activated N-type Ca^2+^ currents in primary cultures of hippocampal cells in a dose-dependent way, displaying an IC_50_ value (0.16 μM) in the same order as that of MVIIA (IC_50_ = 0.20 μM). The blockade effects of SO-3 and MVIIA on N-type calcium channels were both reversible. P/Q- and R- types were not inhibited [111].

ω-Conotoxin TxVII is a L-type Ca^2+^ channel antagonist and ω-conotoxin MVIIC, besides producing a complete N-type channel blockade, also blocks P-type channels in cerebellar Purkinje cells [115].

### 6.2. Sodium Channels

Sodium channels consist of three protein subunits (α, β-1 and β-2) in a 1:1:1 stoichiometry. There are three different types of brain Na^+^ channel α-subunits (I, II, and III) [109]. Based on their susceptibility to be blocked by tetrodotoxin, Na^+^ channels can be divided into tetrodotoxin-sensitive and tetrodotoxin-resistant ones. The first class includes the neuronal type I/Nav1.1, type II/Nav1.2, type III/Nav1.3, PN1/Nav1.7 and PN4/Nav1.6, all of them present in the CNS [116]. Some of these subtypes have been implicated in clinical conditions, such as neuropathic pain [117,118,119], stroke [120] and epilepsy [121].

δ-Conotoxins are known to inhibit the fast inactivation of voltage-gated sodium channels [113]. δ-Conotoxin SVIE (from *Conus striatus* L.) is a strong excitotoxin when injected intracranial on mice [122]. It induced twitching of hind limbs at 12 pmol/g and at higher concentrations (70 pmol/g); SVIE induced more severe excitatory symptoms (running in circles and spastic paralysis). This toxin is more potent than δ-conotoxins PVIA and TxVIA, which did not cause any behaviour changes at 20 pmol/g and 1000 pmol/g, respectively. SVIE (IC_50_ = 12 nM), as well as δ-conotoxins PVIA and TxVIA, was able to displace ^125^I-δ-conotoxin TxVIA in sagittal sections of rat brain.

Sea anemones possess specialized structures, called tentacles, containing a wide variety of toxins that are used in the capture of prey, as well as for defence against predators [123]. APE 1–1 and APE 1–2 (5 μg/mL, each), polypeptides present in the venom of the sea anemone *Anthopleura elegantissima* Brandt, did not affect Na^+^ current activation, but provoked delayed and incomplete inactivation of the current passing through fast Na^+^ channels in mouse neuroblastoma N1E-115 cells [124].

Microinjection of granulitoxin (8 μg), a neurotoxin from sea anemone *Bunodosoma granulifera* Lesueur, into the dorsal hippocampus (CA1–CA3 areas) of rats induced seizure activity and the rats presented behavioural alterations similar to the pilocarpine model of temporal lobe epilepsy: akinesia, facial automatisms, head tremor, salivation, rearing, jumping, barrel-rolling, wet dog shakes and forelimb clonic movements [125].

On the other hand, μ-conotoxins are peptide inhibitors of voltage-sensitive Na^+^ channels. They act selectively to occlude the pore of the channel by competing with tetrodotoxin and saxitoxin [116]. μ-Conotoxin PIIIA, from *Conus purpurascens* G. B. Sowerby II, reduced tetrodotoxin-sensitive voltage-dependent Na^+^ current in rat peripheral and CA1 neurons. In the radioligand binding studies, PIIIA showed the highest potency at rat and human brain voltage-sensitive Na^+^ channels, GIIIB (from *Conus geographus*) exhibited intermediate potency, and GIIIA and GIIIC (from *Conus geographus*) were the less active. However, none of them were able to fully displace [^3^H]saxitoxin from rat or human brain, compared with the displacement induced by tetrodotoxin [116].

### 6.3. Potassium Channels

The human genome encodes 40 voltage-gated K^+^ channels (KV), which are involved in several physiological processes, namely repolarization of neuronal and cardiac action potentials, regulation of Ca^2+^ signalling and cell volume, cellular proliferation and migration. The subtypes present in the CNS are Kv1.1–Kv1.8, Kv2.1, Kv2.2, Kv3.1- Kv3.4, Kv4.1–4.3, Kv7.2, Kv7.3, Kv7.5, Kv10.1, Kv10.2 and Kv11.2 [126].

κ-Conotoxins are antagonists of potassium-gated channels [113]. A κ-conotoxin from *Conus virgo* L., ViTx, inhibited homomeric vertebrate K^+^ channels Kv1.1 (rat; IC_50_ = 1.59 μM) and Kv1.3 (human; IC_50_ = 2.09 μM), but not Kv1.2 (rat) expressed in *Xenopus* oocytes, whereas the κ-conotoxin PVIIA, which blocks the Shaker K^+^ channel, was effective at nanomolar concentration (IC_50_ about 70 nM) [127].

κM-conotoxin RIIIK from *Conus radiatus* Gmelin (4 nmol) administered by intracerebrovascular route into mice caused seizures. However, when the peptide was injected intraperitoneally, there were no visible effects. RIIIK was also shown to inhibit the Shaker K^+^ channel expressed in *Xenopus* oocytes (IC_50_ = 1.21 µM), leading to the hypothesis that RIIIK targets a K^+^ channel subtype in peripheral axons and in combination with other excitatory peptides (such as the δ-conotoxins that inhibit Na^+^ channel inactivation) causes a massive depolarization of peripheral axons near the venom injection site. This elicits bidirectional propagated action potentials, which allow the toxins to cross the BBB and the effect is equivalent to a tonic/clonic seizure, resulting in a very rapid tetanic paralysis of the prey [128].

Marine drugs also modulate ligand-gated ion channels, such as ACh, glutamate, serotonin, histamine, GABA, glycine and norepinephrine receptors.

### 6.4. ACh Receptors

ACh acts on the nervous system through two types of receptors: muscarinic (mAChRs) and nicotinic (nAChRs). Five mAChR subtypes (m1–m5) have been identified, all of them present in the brain. They belong to the superfamily of G-protein-coupled receptors and they trigger second messenger cascades. nAChRs are ligand-gated ion channels that modulate the fast synaptic transmission of ACh and have been implicated in attention, memory, learning, development, antinociception, nicotine addiction, PD, AD, Tourette’s syndrome, certain forms of epilepsy and schizophrenia. nAChRs are mainly located pre-synaptically, but also post-synaptically throughout the CNS [129,130,131]. Pre-synaptic nAChRs regulate the synaptic release of ACh and also of other important neurotransmitters, such as dopamine (DA), norepinephrine (NE), serotonin (5-HT), glutamate (Glu), and γ-aminobutyric acid (GABA), being important targets for the treatment of pain, epilepsy and of a wide range of neurodegenerative and psychiatric disorders. There are 17 identifed genetically distinct subunits of nAChRs, from which 5 are muscle-type (α1, β1, δ, γ and ε) and 12 are neuronal-type (α2–α10 and β2–β4). A functional nAChR comprises five homopentamer or heteropentamer subunits placed symmetrically around a central cation-channel pore. α7 and α4β2 are the most abundant combinations in CNS [129,132,133,134]. The distribution of nAChRs types in CNS was reviewed by Gotti *et al.* [135].

**Figure 4:**
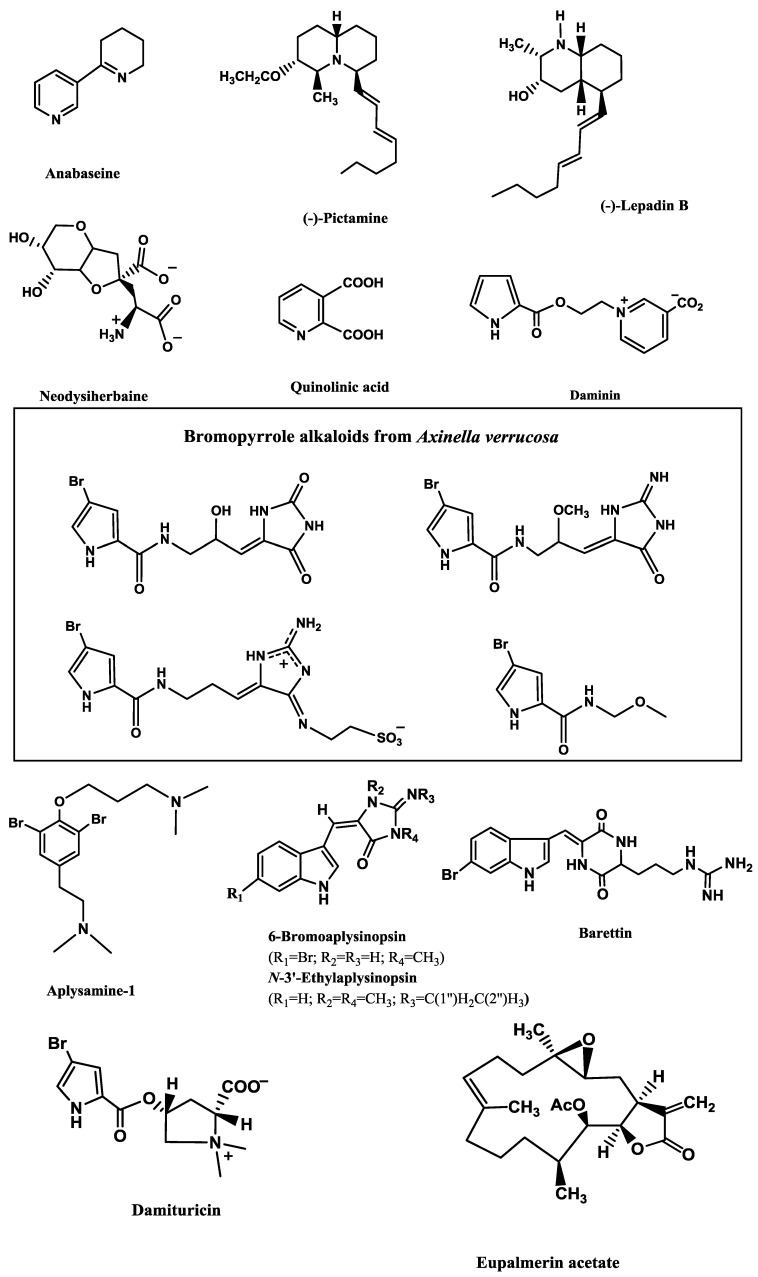
Potent marine modulators of voltage-dependent and ligand-gated ion channels.

Anabaseine (Figure 4), an alkaloid isolated from carnivorous marine worms of the phylum Nemertea, is a non-selective nicotinic agonist. It is a full agonist of *Xenopus* oocyte-expressed rat nAChR α7 receptor, but only a very weak agonist of the α4β2 subtype [136].

On the other hand, several marine drugs have demonstrated inhibition of ACh-elicited current nAChRs (Table 10) expressed in *Xenopus* oocytes [132,134,137,138,139,140,141].

**Table 10:** Marine drugs as nAChR antagonists.

Compound/Organism	nAChR subtype	IC_50_ (nM)
(−)-Lepadin B/ascidian *Clavelina lepadiformis* Müller	α7	0.7 * [132]
(−)-Pictamine/ascidian *Clavelina picta* Verrill	α7	1.3 * [132]
α-conotoxin GID/ *Conus geographus* L.	α7	4.5 [137]
α-Conotoxin ImII/ *Conus imperialis* L.	α7	441.0 [138]
α-Conotoxin ImI/ *Conus imperialis* L.	α7	191.0 [138]
αD-contoxin VxXIIB/ *Conus vexillum* Gmelin	α7	0.4 [134]
α-conotoxin Qc1.2/ *Conus quercinus* Lightfoot	α3β2	<10.0 * [139]
α-conotoxin GID/ *Conus geographus* L.	α3β2	3.1 [137]
α-conotoxin Qc1.2/ *C. quercinus* Lightfoot	α3β4	>10.0 * [139]
(−)-Lepadin B/ascidian *C. lepadiformis* Müller	α4β2	0.9 * [132]
(−)-Pictamine/ascidian *C. picta* Verrill	α4β2	1.5 * [132]
α-conotoxin GID/ *C. geographus* L.	α4β2	152.0 [137]
αD-conotoxin VxXIIB/ *C. vexillum* Gmelin	α3β2	8.4 [134]
αD-conotoxin VxXIIB/ *C. vexillum* Gmelin	α4β2	228.0 [134]
αD-conotoxin VxXIIA/ *C. vexillum* Gmelin	α3β2	370.0 [134]
*Phycotoxins found in marine invertebrate glands*		
13-Desmethyl spirolide C	α7	0.4 [140]
Gymnodiamine	α7	2.0 [140]
13-Desmethyl spirolide C	α4β2	0.7 [140]–3.9 [141]
Gymnodimine	α4β2	0.5 [140]–0.9 [141]

* µM.

ACh (1 µM)-elicited currents through α4β2 subtype and ACh (100 µM)-elicited currents through α7 subtype were blocked by (−)-pictamine (Figure 4) and (−)-lepadin B (Figure 4), two alkaloids from the ascidians *Clavelina picta* Verrill and *Clavelina lepadiformis* Müller, respectively [132].

α-Conotoxins are a class of nAChRs antagonists [142]. Several works have been conducted with rat or mouse nAChRs expressed in *Xenopus* oocytes. Peng *et al.* [139] showed that the α-conotoxin Qc1.2, from *Conus quercinus* Lightfoot, had little effect on rat neuronal α7 subtype at 1 µM and at 10 µM it blocked ACh (100 µM)-elicited currents in α3β2 and α3β4 nAChR subtypes, but not in α4β2 subtype. Similarly, other α-conotoxin, GID, from *C. geographus* L., strongly inhibited rat α7 and α3β2, was less active as α4β2 antagonist, but was at least 1000-fold less potent at α3β4 and α4β4 receptors [137]. α-Conotoxins ImII and ImI from *Conus imperialis* L. were less active than GID against rat α7 nAChR. Using crude rat brain membranes, only ImI (EC_50_ = 1.56 nM) was able to displace 3–^125^I-α-bungarotoxin (4 nM), a snake toxin that is a classical reversible competitive inhibitor of some nAChR subtypes, such as α7 subtype [138].

αD-contoxin VxXIIB was more potent against α7, α3β2 and α4β2 receptors than VxXIIA and VxXIIC, all of them found in the venom of *Conus vexillum* Gmelin [134].

Despite not being produced by marine invertebrates, some phycotoxins are accumulated in phytoplankton and mollusc digestive glands. Examples are the macrocyclic imines spirolines and gymnodimines, which caused fast neurotoxic death when administered to mice. Indeed, four spirolides, A, B, C, and 20-methyl spirolide G, were toxic to mice by intraperitonneal injection, with LD_50_ values of 37.0, 99.0, 8.0 and 8.0 µg/kg BW, respectively [143]. Spirolines function as brain mAChR and nAChR antagonists, while gymnodimines target muscle and neuronal nAChR [141,143]. Besides inhibiting ACh (25 or 150 µM)-evoked currents in neuronal nAChRs, gymnodimine and 13-desmethyl spirolide C from the dinoflagellate *A. ostenfeldii* were also able to inhibit the nicotine (10 µM)-mediated dopamine release from rat striatal synaptosomes containing both α4β2 and α6* receptors, displaying IC_50_ values of 0.3 and 0.2 nM, respectively [140].

### 6.5. Glutamate Receptors

Glutamate (l-Glu) and aspartate are excitatory neurotransmitters in the CNS. They cause excitotoxicity by hyperactivating post-synaptic glutamate receptors, which is observed in ischemia, hypoglycemia, epileptic seizures and in neurodegenerative diseases, such as AD, Parkinsonism, amyotrophic lateral sclerosis and Huntington’s disease. Additionally, pre-synaptic glutamate receptors can modulate neurotransmitter release. There are two types of receptors: ionotropic (ligand-gated cation channels) and metabotropic (G-protein coupled) receptors. Within ionotropic receptors, the three major types are *N*-methyl-d-aspartate (NMDA) receptors (NR1, NR2A-D and NR3A-B), α-amino-3-hydroxy-5-methyl-4-isoazolepropionic acid (AMPA) receptors (GluR1–4) and 2-carboxy-3-carboxymethyl-4-isopropenylpyrrolidine (kainate) receptors (GluR5–7 and KA1–2) [144,145,146,147]. The excitotoxic effect of the receptor agonists is associated with the massive entry of Ca^2+^ into the cells, inducing multiple cytotoxic damage to the neurons, such as perturbation of cytoskeletal proteins and activation of proteases and phospholipases [144,145,148]. However, under physiological conditions, glutamate offers a beneficial effect on the regulation of neuronal function, growth and differentiation [145].

Neodysiherbaine (Figure 4; 50 μM), isolated from the marine sponge *Dysidea herbacea* Keller, is an agonist of AMPA (GluR4) and of kainate (GluR5, GluR6, KA2) receptors [146] and an extract obtained from the marine sponge *Suberites domuncula* Olivi containing quinolinic acid (Figure 4) is an agonist of NMDA receptors [149].

Two important marine toxins that also bind KA and AMPA receptors and provoke excitotoxicity are kainic and domoic acids, algae-derived metabolites that can accumulate in shellfish. Both acids are potent agonists of kainate and AMPA subclasses of Glu-receptors [150]. Doucette *et al.* [151] tested the toxicity of these two toxins in neonatal rats. Domoic acid proved to be more toxic than kainic acid (ED_50_ = 0.08 and 0.43 mg/kg at postnatal day 8 and ED_50_ = 0.19 and 1.19 mg/kg at postnatal day 14).

On the other hand, several antagonists of glutamate were isolated from marine invertebrate organisms. Aiello *et al.* [152] incubated rat primary cortical cells with 200 μM of l-Glu and 2.4 mM CaCl_2_, which resulted in a strong rise in [Ca^2+^]i. However, incubation of daminin (Figure 4; 0.5, 1.0 and 3.0 μg/mL), a bioactive pyrrole alkaloid from the sponge *Axinella damicornis* Esper, resulted in a significant decrease of [Ca^2+^]i. Moreover, this alkaloid (1.0 μg/mL) also reverted the increase of [Ca^2+^]i induced by 200 μM of NMDA and 2.4 mM CaCl_2_. A similar neuroprotective effect was found for four bromopyrrole alkaloids (Figure 4) characteristic of the sponge *Axinella verrucosa* Esper [145]. Pre-incubation of rat neurons with 10 μg/mL of these compounds counteracted the increase in [Ca^2+^]i provoked by 200 μM L-Glu and 2.5 mM CaCl_2_. Moreover, they also decreased the rise of free [Ca^2+^]i induced by 200 μM quisqualic acid (QUIS), a selective agonist of the metabotropic glutamate receptors, and by 2.5 mM CaCl_2_.

The peptides conantokins are NMDA receptor antagonists present in *Conus* venoms and are currently being tested as potential anticonvulsants. Jimenez *et al.* [153] showed that, although conantokin-L appears to be almost as potent as conantokin-R in NMDA receptor binding assays, the last is a more potent anticonvulsant compound, with a protective index of 17.5 (*vs.* 1.2 for conantokin-L) when tested in the audiogenic mouse model of epilepsy. Furthermore, conantokin-R was 2–5 times more effective (IC_50_ = 93 nM) than conantokin-G or conantokin-T as NMDA receptor antagonist, in the assay involving inhibition of binding of the non-competitive antagonist of the NMDA receptor, [^3^H]MK-801, to the NMDA receptors in rat brain membranes [154]. Anyway, conantokin-G showed neuroprotection in a rat model of focal cerebral ischemia, when delivered intrathecally, and its protection lasted for 8 h [155].

### 6.6. Serotonin Receptors

Serotonin (5-HT) is a neurotransmitter that plays an important role in normal brain function and modulation of sleep, mood, appetite, sexual function, memory, among others. This neurotransmitter binds to different subtypes of serotonin receptors (5HT_1_–5HT_7_). 5HT_3_ receptor is the only class of ligand-gate ion channels, while the others are G protein-coupled receptors [14].

Hu *et al.* [156] isolated twelve compounds from the sponge *Smenospongia aurea* Pulitzer-Finali, which included the sesquiterpenes aureol, 6′-chloroaureol and aureol acetate, and the alkaloids 3-carboxylindole, *N*,*N*-dimethyltryptamine, isoplysin A, 2′-de-*N*-methyl-aplysinopsin, 6-bromo-2′-de-*N*-methylaplysinopsin, 6-bromoaplysinopsin (Figure 4), *N*-3′-methylaplysinopsin and *N*-3′-ethylaplysinopsin (Figure 4). In the radioligand binding assays of crude membranes, only 6-bromo-2′-de-*N*-methylaplysinopsin (*Ki* = 2.3 µM), 6-bromoaplysinopsin (*Ki* = 0.3 µM) and *N*-3′-ethylaplysinopsin (*Ki* = 3.5 µM) displaced high-affinity [^3^H]mesulergine binding from cloned human 5-HT_2C_ receptors. The last two compounds also displaced [^3^H]methylspiperone from 5-HT_2A_ subtype (*Ki =* 2.0 and 1.7 µM, respectively). Structure–activity analysis of these aplysinopsins revealed the importance of the functional groups at positions 6, 2′ and 3′ to bind to the receptors. The length of the alkyl chain at 3′ is a key factor, since the active *N*-3′-ethylaplysinopsin differs in one CH_3_ group in relation to the inactive *N*-3′-methylaplysinopsin. When ethylation is not present, 6-bromination contributes to the binding activity and is also important for selective binding to the 5-HT_2C_ receptor subtype. Moreover, methylation in position 2’ contributes for the selectivity towards 5-HT_2A_ receptors.

Hedner *et al.* [157] tested two brominated cyclodipeptides from the sponge *Geodia barrette* Bowerbank for binding different subtypes of 5-HT receptors expressed in HEK-293 cell membranes (5-HT_1A_, 5-HT_1D_, 5-HT_2A_, 5-HT_2C_, 5-HT_3A_, 5-HT_4,_ 5-HT_5A,_ 5-HT_6_ and 5-HT_7A_). 8,9-Dihydrobarettin had affinity only for 5-HT_2C_ (*Ki* = 4.63 µM), while barettin binded to 5-HT_2A_ (*Ki* = 1.93 µM), 5-HT_2C_ (*Ki* = 0.34 µM) and 5-HT_4 _(*Ki* = 1.91 µM). Barettin (Figure 4) clearly has its advantages at the 5-HT_2C_ receptor, with a selectivity ratio of 5.68 (5-HT_2A_/5-HT_2C_) between the two 5-HT_2_ receptor subtypes. The small difference between barettin and 8,9-dihydrobarettin, which differ in one double bond in the tryptophan residue, greatly affected the affinity.

Two bromopyrrole alkaloids, damipipecolin and damituricin (Figure 4), from the sponge *Axinella damicornis* Esper, displayed a modulating effect of serotonin receptor activity *in vitro*. The marked increase of [Ca^2+^]i observed in primary neural cells under the effect of 200 µM 5-HT and 2.5 mM CaCl_2_ was strongly reduced by damipipecolin (0.1 µg/mL) or damituricin (0.1 µg/mL). However, only damituricin displayed the same behaviour in PC12 cells, revealing that it is a strong 5-HT_3_ antagonist [158].

### 6.7. Histamine (H_3_) Receptor

There are four types of histamine receptors, namely H_1_, H_2_, H_3_ and H_4_. H_3_ receptor is an attractive G protein-coupled receptor drug target that modulates neurotransmission in the CNS and plays a role in cognitive and homeostatic functions. H_3_ receptors are located pre-synaptically and their antagonists regulate sleep, food intake and obesity, memory, spatial recognition, attention, impulsivity, psychosis, seizures and depression, since they have a direct effect on neurotransmitters’ release, particularly acetylcholine, noradrenaline and dopamine. Therefore, this receptor is an attractive CNS drug target [159,160].

Aplysamine-1 (Figure 4), a bromotyrosine derived metabolite isolated from the sponge *Aplysina* sp., was found to possess a high binding affinity for the human H_3_ receptor (*Ki* = 30 ± 4 nM). The human and rat binding affinities were determined for aplysamine-1 and a series of analogs. Structure–activity relationship analysis examined three regions, the bromo-substituent effect, the alkoxy and alkyl amine chain lengths and the size of the two amine groups. Increases are observed when the removal of the aryl bromines or the replacement of the dimethylamine on the alkoxy chain with a piperidine occurs [160].

### 6.8. GABA_A_ Receptor

The ionotropic γ-aminobutyric acid receptors (GABA_A_R) are a member of the superfamily of ligand-gated ion channels sharing many structural and functional features with the nicotinic receptor. The GABA_A_R mediates the major component of fast inhibitory transmission in the CNS, and potentiators of the GABA_A_R can act as anxiolytics, anticonvulsants, hypnotics, tranquillizers or anaesthetics [161]. GABA_A_R are pentameric heteromers assembled from 5 of 19 subunits (six α, four β, three γ, one δ, one ɛ, one π, and three ρ subunits), each encoded by different genes [162].

Eupalmerin acetate (Figure 4) is a marine diterpene compound isolated from the gorgonian octocorals *Eunicea succinea* Pallas and *Eunicea mammosa* Lamouroux. This compound dose-dependently (3 or 30 µM) potentiated macroscopic currents elicited by GABA (5 µM) or pentobarbital (100 µM) in HEK cells expressing α1β2γ2L, displaying an EC_50_ of 17.4 µM. This potentiation was reduced when 1 mM of GABA was applied. Single-channel experiments were conducted with GABA (50 µM) and eupalmerin acetate (40 µM) and revealed that the diterpene was mechanistically similar to neurosteroids and probably interacts with the steroid-binding site. Indeed, (3α,5α)-17-phenylandrost-16-en-3-ol, an antagonist of neurosteroids potentiation, but not of barbiturates and benzodiazepines, reduced the effect of eupalmerin acetate in HEK cells [161].

### 6.9. Glycine Receptors

Glycine-gated chloride channel receptors (GlyRs) are members of ligand-gated ion channels family comprising subunits α1–α4 and β. As GABA_A_R, they are key modulators of inhibitory neurotransmission in CNS. GlyRs are formed either as pentameric homomers or as αβ heteromers [163]. Potentiators and antagonists of glycine receptors are listed in Table 11 [163,164,165].

**Table 11:** Marine drugs as GlyR modulators.

Compounds/Organisms	GlyR subtype	Inhibition (IC_50_), μM	Potentiation (EC_50_), μM
8-Hydroxyircinialactam B/sponge *Sarcotragus* sp.	α1	0.5 [164]	-
8*E*-3′-Deimino-3′-oxoaplysinopsin + 8*Z*-3′-deimino-3′-oxoaplysinopsin/sponge *Lanthella flabelliformis* Pallas	α1	>200 [163]	-
Tubastrindole B/sponge *L. flabelliformis* Pallas	α1	25.9 [163]	-
(−)-Ircinianin sulfate/sponge *Psammocinia* sp.	α1	38.4 [165]	-
(12 *E*,20*Z*,18*S*)-8-Hydroxyvariabilin/sponge *Ircinia variabilis*	α1	-	1.2 [164]
8 *E*-3′-Deimino-3′-oxoaplysinopsin + 8*Z*-3′-deimino-3′-oxoaplysinopsin/sponge *L. flabelliformis* Pallas	α3	67 [163]	-
(12 *E*,20*Z*,18S)-8-hydroxyvariabilin/sponge *I. variabilis*	α3	7.0 [164]	-
(−)-Ircinianin sulfate/sponge *Psammocinia* sp.	α3	3.2 [165]	-
Ircinialactam A/sponge *Sarcotragus* sp.	α3	30–100 [164]	-
Ircinialactam C/sponge *Sarcotragus* sp.	α3	30–100 [164]	-
Ent-ircinialactam C/sponge *Sarcotragus* sp.	α3	30–100 [164]	-
Ircinialactam D/sponge *Sarcotragus* sp.	α3	30–100 [164]	-
Tubastrindole B/sponge *L. flabelliformis* Pallas	α3	>300 [163]	-
Ircinianin lactam A/sponge *Psammocinia* sp.	α3	-	8.5 [165]

Balansa *et al.* [163] isolated two new sesquiterpene glycinyl lactams, ianthellalactams A and B, the sesquiterpene dictyodendrillin and its ethanolysis artifact ethyl dictyodendrillin, and five indole alkaloids, aplysinopsin, 8*E*-3′-deimino-3′-oxoaplysinopsin, 8*Z*-3′-deimino-3′-oxoaplysinopsin, dihydroaplysinopsin and tubastrindole B. They also synthetized alkaloid analogs to establish a relationship between the structure and the inhibitory activity towards GlyR. They concluded that conversion of 3′-imino to 3′-oxo moiety and the increase of *N*-methylations led to an increase of inhibition.

### 6.10. Norepinephrine Transporter (NET)

χ-Conopeptide MrIA and MrIB from *Conus marmoreus* L. inhibited the norepinephrine transporter [166,167]. χ-MrIA inhibited the binding of [^3^H]nisoxetine to the membranes of cells expressing the rat and human NET. The IC_50_ for inhibition was 500 nM for the rat NET and 1.7 μM for the human NET. [^3^H]mazindol binding to the expressed transporters was also sensitive to χ-MrIA, exhibiting IC_50_ values of 1.9 μM at the rat NET and of 4.0 μM at the human NET. In cells transfected with the human NET, MrIA became a less effective blocker of [^3^H]norepinephrine under reduced extracellular Na^+^ conditions [166].

## 7. Protective Effect of Marine Drugs Using Cell Models for Neurodegenerative Disorders

Although some cnidarian venoms, such as those isolated from the nematocysts of the jellyfish *Pelagia noctiluca* Slabber, induce oxidative stress on neuronal-like cells derived from human neuroblastoma SH-SY5Y, by disrupting mitochondrial membrane potential [168], several marine drugs have shown protective effect on several cell models for neurodegenerative diseases.

### 7.1. Protection against Aβ-Induced Neurotoxicity

Aβ peptide induces protein oxidation, lipid peroxidation and reactive oxygen species (ROS) formation in AD patients’ brains [54]. Neuronal dysfunction in AD may occur before the deposition of insoluble fibrillar Aβ and seems to be mediated by soluble Aβ oligomers [56,169]. Peptides with shorter sequences, such as Aβ25–35, can also result from certain forms of Aβ1–40. This short peptide has been reported to be more soluble and easier to inject *in vivo* than Aβ1–40, as it is more toxic and causes more oxidative damage [56,84].

**Figure 5:**
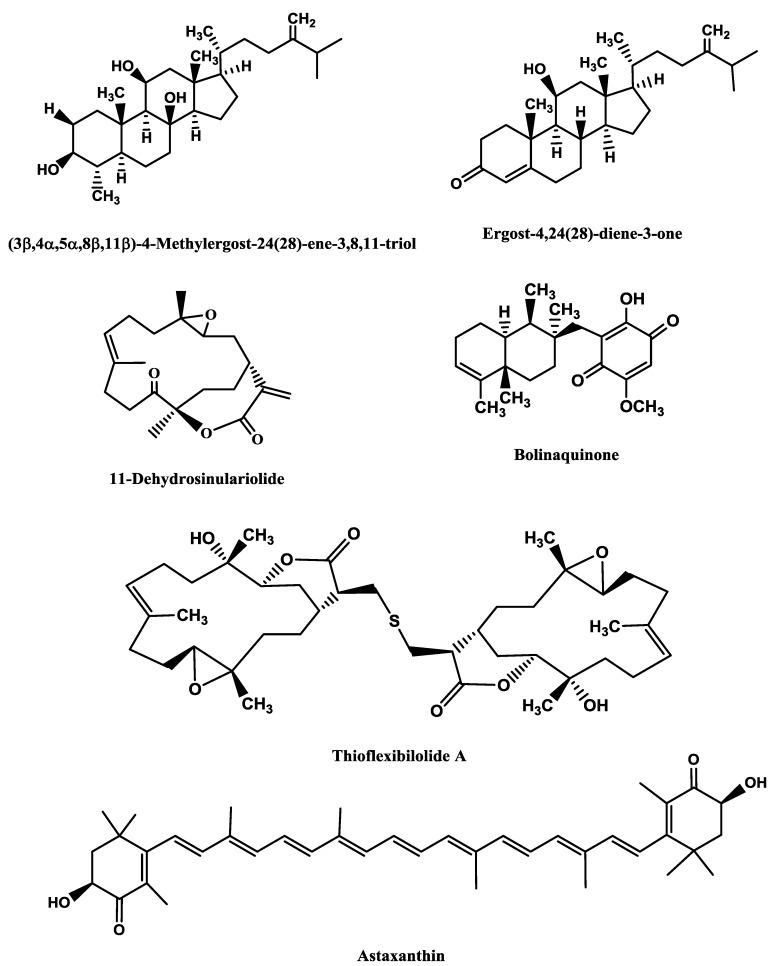
Marine compounds with protective effects against Aβ, 6-OHDA, MPP^+^ and IAA.

The steroids (3β,4α,5α,8β,11β)-4-methylergost-24(28)-ene-3,8,11-triol (Figure 5) and ergost-4,24(28)-diene-3-one (Figure 5), from the soft coral *Sinularia depressa* Tixier-Durivault, at 10 μM, displayed neuroprotective effects against Aβ25–35 (10 μM)-induced cellular injuries in SH-SY5Y cells and induced the increase of cell viability by 20.1% and 16.6%, respectively [170].

### 7.2. Protection against 6-Hydroxydopamine (6-OHDA)-Induced Neurotoxicity

The neurotoxin 6-OHDA is a hydroxylated analog of dopamine, commonly used to study dopaminergic degeneration, both *in vitro* and *in vivo*. Like DA, 6-OHDA quickly oxidizes to form ROS, including hydrogen peroxide (H_2_O_2_), superoxide (O_2_^•−^) and hydroxyl radicals (•OH) [61,171,172]. This neurotoxin also reduces striatal glutathione (GSH) and superoxide dismutase (SOD) enzyme activities and increases the level of malondialdehyde [173,174]. Besides causing oxidative stress, 6-OHDA also leads to respiratory inhibition, as it is toxic to the mitochondrial complex I [175]. Both mechanisms are not necessarily linked, but appear to act synergistically during neuron degeneration. However, 6-OHDA model does not mimic all pathological and clinical features of human Parkinsonism, because it induces dopaminergic neuron death with preservation of non-dopaminergic neurons, without formation of cytoplasmic inclusions (Lewy bodies). Moreover, 6-OHDA does not affect other brain areas involved in PD, and Parkinsonian-like tremor is rare in studies of 6-OHDA-lesioned rodents [60].

11-Dehydrosinulariolide (Figure 5), a terpenoid obtained from the marine soft coral *Sinularia flexibilis* Quoy and Gaimard, displayed protective effects against 6-OHDA (20 μM)-induced cytotoxicity in SH-SY5Y cells, at concentrations ranging from 1 nM to 1 μM. Moreover, pre-treatment with 11-dehydrosinulariolide (10 nM) also inhibited the down-regulation of phospho-Akt protein expression induced by 6-OHDA, as well as inhibited 6-OHDA-induced caspase-3/7 activation and 6-OHDA-induced translocation of NF-κB to the nucleus. 11-Dehydrosinulariolide (10 nM) inhibited the down-regulation of p-ERK induced by 6-OHDA [176]. The PI3K–Akt and ERK (p42/p44 mitogen-activated protein kinase) pathways are important factors in neuronal cell survival. Their activation was suggested to have neuroprotective effects in PD [176]. AKT, a Ser/Thr protein kinase, regulates a variety of cellular processes, including cell survival, proliferation, protein translation and metabolism [177]. PI3K pathway can activate the kinase Akt, which is also implicated in cell survival, proliferation and growth, as well as in glycogen metabolism [178]. NF-κB is an inducible transcription factor that plays an important role in human inflammatory processes and various neurodegenerative diseases [179,180]. Moreover, the same authors [176] verified the *in vivo* effects of 11-dehydrosinulariolide, which was able to significantly attenuate the 6-OHDA-induced reduction of mean swimming velocity and total swimming distance in zebrafish.

A similar result was found for the sulfur-containing biscembranolide thioflexibilolide A (Figure 5), isolated from the same soft coral. Thioflexibilolide A exhibited neuroprotective activity against 6-OHDA in SH-SY5Y cells between 0.001 and 10 µM, displaying relative neuroprotective effect of 37.2 (at 0.001 µM) and 73.2% (at 0.01 µM), though it decreased for higher concentrations [181].

In a study developed by Ikeda *et al.* [172], the treatment with 6-OHDA (100 µM) markedly induced apoptosis in SH-SY5Y cells by 2.8-fold, but a pre-treatment with astaxanthin (Figure 5; 1–20 µM) significantly suppressed apoptosis in a dose-dependent manner (6%–54% inhibition). Astaxanthin (5–20 µM) also dose-dependently suppressed the cleavage of caspase 3 and of poly(ADP-ribose) polymerase (PARP) induced by 6-OHDA (100 µM), indicating that this compound inhibited caspase-3 activation, as well as caspase 3 activity by 14% (5 µM), 40% (10 µM), and 49% (20 µM). Astaxanthin (20 µM) also displayed protective effect against 6-OHDA (100 µM)-induced mitochondrial dysfunctions, since it significantly increased membrane potential (ΔΨm), protected cytochrome c and inhibited caspase 9 cleavage, which is triggered by mitochondrial dysfunction. In addition, 6-OHDA (100 µM) induced both p38 MAPK and ERK1/2 activation, whereas astaxanthin (20 µM) blocked the activation of p38 MAPK, but not of JNK1/2 or ERK1/2. Like astaxanthin, pre-treatment with SB203580 (20 µM), a specific inhibitor of p38 MAPK, also displayed the same protective effects against mitochondrial dysfunction. Finally, a pre-treatment with astaxanthin (5–20 µM) also significantly decreased 6-OHDA-induced ROS generation in a dose-dependent manner (11, 41 and 55% inhibition at 5, 10, and 20 µM, respectively) [172].

### 7.3. Protection against 1-Methyl-4-Phenyl-Pyridine Ion (MPP+)-Induced Neurotoxicity

The model using the dopaminergic neurotoxin 1-methyl-4-phenyl-1,2,3,6-tetrahydropyridine (MPTP), an analog of the narcotic meperidine, causes intoxication of dopaminergic structures and induces symptoms resembling PD in humans. MPTP is highly lipophilic and after systemic administration rapidly crosses the BBB. Afterwards, this toxin is converted to 1-methyl-4-phenyl-2,3-dihydropyridium (MPDP) in non-dopaminergic cells (mainly in astrocytes and serotonergic neurons) by the enzyme monoamine oxidase B (MAO-B) and then spontaneously oxidizes to MPP^+^. This polar molecule enters dopaminergic cells through carrier systems and causes oxidative stress. MPP^+^ inhibits the mitochondrial complex I, causing abnormal energy metabolism and increased ROS (O_2_^•−^, H_2_O_2_ and •OH) production, resulting in lipid peroxidation, DNA fragmentation, mitochondrial impairment, LDH leakage, GSH depletion, reduction of Na^+^/K^+^-ATPase and catalase activities, increased caspase-3 activity and cell death [60,182].

Astaxanthin (Figure 5; 10 and 20 µM) showed neuroprotective effects against the neurotoxin MPP^+^ (500 µM) in PC12 cells, increasing cell viability by 3.46%. Moreover, mithramycin A (0.36 µM), a specific SP1-DNA binding inhibitor, increased viability by 34.94%, and a co-treatment with mithramycin A (0.36 µM) plus astaxanthin (10 µM) increased cell survival by 26.77%. During oxidative stress, the transcription activator Sp1 is up-regulated, leading to up-regulation of NMDA receptor subunit 1 (NR1), which initiates neuronal cell death. Expression of Sp1 and NR1 protein levels in the MPP^+^ group increases and Sp1 is transferred from nuclei to cytoplasm, but this effect is also reverted by mithramycin A and/or astaxanthin. MPP^+^ (500 µM) treatment led to an increase of ROS activity by 26.14%, but astaxanthin induced ROS activity to decrease by 4.75% at 5 µM, 9.36% at 10 µM, 14.60% at 20 µM. Mithramycin A (0.36 µM) only provoked a decrease of 8.79% [182].

### 7.4. Protection against Iodoacetic acid (IAA) Neurotoxicity

IAA induces cell death following depletion of intracellular ATP, mitochondrial dysfunction and production of ROS. Since these observations are similar to those of *in vivo* ischemic stroke, this is a good cell model to study this disease [38].

The neuroprotective effects of dysideamine (Figure 1) and bolinaquinone (Figure 5), sesquiterpene quinones isolated from the marine sponge *Dysidea* sp. 05C33, against IAA-induced cell death were examined. At 10 µM, both compounds exhibited neuroprotective effect against IAA-induced cell death (43 and 57% of cell survival for dysideamine and bolinaquinone, respectively). The IAA (10 or 20 µM)-treated mouse hippocampal neuronal cells HT22 showed depletion of intracellular ATP, mitochondrial dysfunction and increase of ROS production, which was inhibited by dysideamine (10 µM) [38].

## 8. Anti-Neuroinflammatory Activity of Marine Drugs

Neuroinflammation is a complex process involved in the pathology of several CNS diseases, such as AD, PD, multiple sclerosis and ischemic stroke, and involves activated microglia [183,184]. Activated microglial cells activate inflammatory mediators, such as proteolytic enzymes [185], ROS and reactive nitrogen species [183,184,185,186], eicosanoids [186,187], pro-inflammatory cytokines [185,186,188] and chemokines [185,186,189], which can promote nociceptive transmission by causing activation of dorsal horn neurons. Many studies have indicated that inhibition of microglial activation attenuates the development of neuropathy [183].

Two COX isozymes, COX-1 and COX-2, catalyse the rate-limiting steps of eicosanoids (prostaglandin (PG) and thromboxane) synthesis, by converting arachidonic acid into PGG2 and PGH2 and then into PGE2, PGF2α, PGD2, PGI2 and tromboxanes (TXB2) [187]. Prostaglandins are critically involved in peripheral and spinal nociceptive sensitization. In general, COX1 is considered to be constitutive, while COX2 is considered as inducible, especially under inflammatory conditions. In the brain, COX2 is constitutively expressed only by specific neuronal populations, particularly in the hippocampus, being necessary for synaptic plasticity and memory acquisition. Inhibition of COX-2, but not of COX-1, by selective inhibitors attenuates hyperalgesia in neuropathic rats [190]. Moreover, although nitric oxide (•NO) acts as cellular messenger and modulates neurotransmition, its overproduction has been associated with neuropathological disorders, such as stroke, AD and PD [191]. Therefore, COX 1 and COX 2, as well as the enzyme neuronal nitric oxide synthase (nNOS), responsible for the synthesis of •NO, represent important therapeutic targets for the development of novel anti-neuroinflammatory drugs.

**Figure 6:**
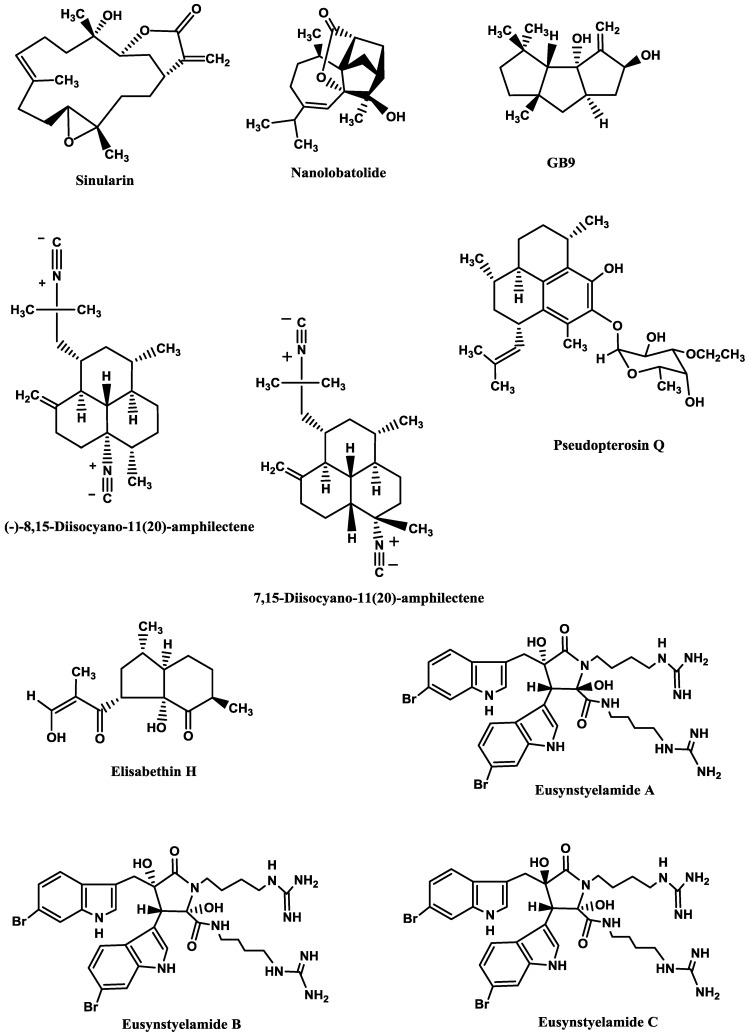
Anti-neuroinflammatory marine compounds.

Sinularin (Figure 6), a cembranolide diterpene isolated from the soft coral *Sinularia querciformis* Pratt, displayed *in vitro* anti-inflammatory activity by significantly inhibiting up-regulation of pro-inflammatory proteins (inducible NOS (iNOS) and COX-2) in LPS-stimulated murine macrophage RAW 264.7 cells. Sinularin (0.1–20 μM) dose-dependently reduced the levels of iNOS and increased those of TGF-β, while COX-2 levels were only reduced at 10 and 20 μM. *In vivo*, subcutaneous administration of sinularin (80 mg/kg, intraplantar) to rats had analgesic effects and inhibited carrageenan-induced spinal neuroinflammation, up-regulation of microglial and astrocyte activation and up-regulation of iNOS in the dorsal horn of the lumbar spinal cord. Furthermore, treatment with sinularin (80 mg/kg) clearly inhibited carrageenan-induced leukocyte infiltration and up-regulated TGF-β1, demonstrating its analgesic effect [192]. Nanolobatolide (Figure 6), a C_18_ terpene from the soft coral *Sinularia nanolobata* Verseveldt, at 10 µM, also reduced the accumulation of iNOS in microglial cells stimulated with INFγ to 45.5% [193].

Δ9(12)-Capnellene-8β,10α-diol (Figure 6; GB9), a sesquiterpene isolated from the soft coral *Capnella imbricata* Quoy and Gaimard, was able to down-regulate the expression of pro-inflammatory iNOS (IC_50_ = 17.1 µM) and COX-2 (IC_50_ = 6.21 µM) in INFγ-stimulated mouse microglial cells (BV2). Moreover, GB9 revealed an analgesic effect *in vivo*. GB9 (10 mg/kg, intraperitoneal) significantly inhibited chronic constriction injury (CCI)-induced thermal hyperalgesia behaviour in rats, as well as inhibited CCI-induced elevation of microglial and neuronal COX-2 in the spinal cord [183].

Diterpene isocyanides isolated from marine sponge *Hymeniacidon* sp. (7-isocyano-11(20)-15(16)-amphilectadiene, (−)-8,15-diisocyano-11(20)-amphilectene (Figure 6), 7,15-diisocyano-11(20)-amphilectene (Figure 6), 8-isocyano-11(20)-ene-15-amphilectaformamide and monamphilectine A) were screened for anti-neuroinflammatory activity in LPS-activated rat brain microglia. They inhibited TXB2 generation (IC_50_ = 0.20–4.69 µM), (−)-8,15-diisocyano-11(20)-amphilectene (IC_50_ = 0.23 µM) and 7,15-diisocyano-11(20)-amphilectene (IC_50_ = 0.20 µM) being the most active ones. However, all demonstrated minimal effect on O_2_^•−^ release (IC_50_ > 10 µM) [184]. Using the same system, Rodriguéz *et al.* [194] and Shi *et al.* [195] tested the anti-inflammatory activity of diterpenoid compounds isolated from the gorgonian *Pseudopterogorgia elisabethae* Bayer. The most promising ones were pseudopterosin Q (Figure 6) (IC_50_ = 4.7 µM against TXB2 and IC_50_ = 11.2 µM against O_2_^•−^ [194] and elisabethin H (Figure 6; IC_50_ = 7.0 μM against O_2_^•−^) [195]. However, due to its cytotoxicity (LDH release > 50% at 3.4 µM), the inhibition of TXB2 by pseudopterosin Q could result, at least in part, from a toxic rather than a pharmacological effect [194].

Finally, the brominated tryptophan-derived eusynstyelamides A, B, and C (Figure 6), isolated from the ascidian *Eusynstyela latericius* Sluiter, exhibited inhibitory activity against nNOS in rat cerebella, with IC_50_ values of 41.7, 4.3 and 5.8 μM, respectively [191].

## 9. Marine Skeletons as Scaffolds for Neural Tissue Engineering

When large tissue volume is lost due to an injury, tissue implantation is advantageous over cell therapy because it enables controlled organization of neurons into intricate networks before implantation [28]. Thus, the aim of bioengineering is to deliver cells and signalling factors to a target tissue in combination with a non-cellular scaffolding material, which is an immobilization matrix that facilitates tissue ingrowth and regeneration [196]. This three-dimensional (3D) cell cultures mimic the cytoarchitecture of *in situ* tissue to a higher degree than cells grown on non-physiological hard surfaces (2D) and, therefore, 3D cultures have been shown to result in longer neurite outgrowth, higher levels of survival and distinct patterns of differentiation as compared to 2D monolayers [197]. An ideal 3D scaffold must not only facilitate the adherence, spread and outgrowth of neurons and neuronal process, but also possess a large number of pores to allow cell expansion and diffusion transport of nutrients and waste molecules to and from the cells. Naturally derived scaffolds (e.g., gels of collagen and chitosan, polysaccharide fibres and aragonite) and synthetic polymers, such as methyl cellulose, poly(α-hydroxyacids), poly(glycolic acid), poly(L-lactic acid) and poly(lactic-co-glycolic acid) are being tested [198,199,200,201].

One of the most efficient scaffolds for neural development is the biodegradable and biocompatible aragonite, a needle-like crystalline form of calcium carbonate (CaCO_3_) present in the exoskeleton of foraminiferans, sponges, corals, hydrozoans, molluscs (gastropods, bivalves, cephalopods), worms, arthropods (ostracods, barnacles). In invertebrates, this skeleton provides mechanical support for the soft tissues and act as a storage system withdrawing ions during times of special physiological demand [196,202,203]. This scaffold presents several advantages over other templates, like hydrogels, not only because its pores are much larger than those of the hydrogels (160 mm *vs.* few microns), allowing many more cells to accumulate, but also because it can release Ca^2+^ to the medium, promoting cell adhesion, cell–cell contact and survival. Moreover, it also provides higher mechanical strength than hydrogels and the absence of a gel covering the cells may facilitate the explant–tissue interactions [202].

Shanny *et al.* [198] grew rat hippocampal primary neurons on aragonite skeleton of the coral *Porites lutea* Link and observed that the neurons usually grew on a sheet of glial cells and acquired the morphology of hippocampal pyramidal and granule neurons. Moreover, dendrites were branched and long, sometimes extending more than 100 µm away from the cell body, and axons were thinner than dendrites and grew up to hundreds of µm in length in all directions, covering the entire surface of the aragonite support. Synaptic connections were active in these neurons, since the presynaptic sites expressed the synaptic vesicle protein 2 (SV2) and post-synaptic spines contained the glutamate receptors GluR1. Peretz *et al.* [202] proved that *P. lutea* aragonite matrix not only was a good support to cell growth *in vitro*, but also *in vivo*. They implanted the scaffolds in cortical regions of postnatal rat brains and observed that the implants did not cause any severe inflammation or rejection response and did not have significant influence on animal survival or behaviour. The implants were invaded by neural tissue and, besides supporting the survival of neurons in the cortex, they induced their invasion into the injured area.

Using a different marine scaffold, Baranes *et al.* [28] grew co-cultures of primary neurons and glia from rat hippocampi on aragonite matrices from the hydrozoan *Millepora dichotoma* Forsskål. Conversely to the *P. lutea* matrix, this scaffold supported ganglion-like cell spheres, rather than multi-layer cells, which included both astrocytes and mature neurons with active synaptic processes. The spheres were interconnected through fibres of neuronal and astrocytic processes and most of the cells had only cell–cell and no cell–matrix interactions. This cell organization resembles more the *in vivo* situation, where neurons do not exhibit substrate contact. Moreover, it has also several advantages for neural tissue engineering, because most cells in the spheres are in contact with other cells, instead of with the matrix surface, and it is easier to detach cells from the scaffold since they are connected to the surface through a neck [28].

*M. dichotoma*-derived aragonite matrix was also used to study the Ca^2+^ uptake by neuronal and glial cells [199]. The authors found that hippocampal cells growing on ^45^Ca^2+^ or calcein-labelled aragonite took up aragonite-derived Ca^2+^ and enhanced this uptake when extracellular Ca^2+^ ions were chelated by EGTA. These ions activate Ca^2+^-dependent adhesion molecules, like cadherins, which play important roles in cell migration, cell rearrangement and maintenance of tissue integrity.

## 10. The Supply Problem

Despite the long research on marine drugs, few of them will be successfully marketed with the current technologies available [204]. This occurs because a sustainable supply of marine organisms is necessary to conduct preclinical and clinical trials. Indeed, the continuous supply problem is the major challenge to be overcome in programs of marine natural product drug discovery and development, in order to move on in the pipeline [205,206]. This problem is reflected in the few number of neuroprotective drugs approved by FDA (see Introduction section), as well as the few studies conducted *in vivo* cited in this review.

Several strategies to overcome the supply problem are being under development, such as sampling strategies, nanoscale NMR for structure elucidation, total chemical synthesis, semi-synthetic production, fermentation, and biotechnology [204,205]. Total synthesis may be the best approach to solve the problem. However, despite all efforts, until now few neuroprotective drugs can be obtained this way. As an example, of 184 compounds reffered to in this review, only a few were already obtained in laboratory. These compounds are listed in Table 12 by the order of appearance in the text.

**Table 12:** Compounds with their total synthesis described.

Compound	Class of compounds	Pharmacologic activity	Ref.
Lembehyne	Linear polyacetylene	Neuritogenic agent	[207]
Turbotoxin A	Diiodotyramine derivative	AChE inhibitor	[67]
Lamellarin O	Alkaloid	BACE1 inhibitor	[208]
Hymenialdisine and analogs	Diterpene isocyanide	Kinase inhibitor	[209,210,211,212]
Lamellarin D and analogs	Alkaloid	Kinase inhibitor	[213,214,215,216]
Lamellarin H	Alkaloid	Kinase inhibitor	[213]
Lamellarin L	Alkaloid	Kinase inhibitor	[215,217]
Lamellarin N	Alkaloid	Kinase inhibitor	[215]
Leucettamine B and analogs	Alkaloid	Kinase inhibitor	[90,218]
Manzamine A	Alkaloid	Kinase inhibitor	[219,220]
Palinurin	Furanoterpenoid	Kinase inhibitor	[221]
Fascaplysin	Alkaloid	Kinase inhibitor	[222,223]
ω-SO-3	ω-Conotoxin	Calcium channels modulator	[224]
δ-SVIE	δ-Conotoxin	Sodium channels modulator	[122]
μ-PIIIA	μ-Conotoxin	Sodium channels modulator	[116]
κM-RIIIK	κM-Conotoxin	Potassium channels modulator	[128]
Anabaseine	Alkaloid	AChR modulator	[225]
(−)-Pictamine	Alkaloid	AChR modulator	[226,227]
(−)-Lepadin B	Alkaloid	AChR modulator	[228,229,230]
α-Qc1.2	α-Conotoxin	AChR modulator	[139]
α-ImI	α-Conotoxin	AChR modulator	[138,231]
α-ImII	α-Conotoxin	AChR modulator	[138]
Spirolide C	Macrocyclic imine	AChR modulator	[232]
Gymnodimine	Macrocyclic imine	AChR modulator	[233]
Neodysiherbaine	Amino acid	Glu receptor modulator	[234,235,236,237]
Daminin	Alkaloid	Glu receptor modulator	[152]
Kainic acid	Amino acid	Glu receptor modulator	[238,239,240,241]
Domoic acid	Amino acid	Glu receptor modulator	[242]
Conatokin-L	Conantokin	Glu receptor modulator	[153,243]
Conatokin-R	Conantokin	Glu receptor modulator	[157]
Aureol	Sesquiterpene	5-HT receptor modulator	[244,245,246]
*N,N*-Dimethyltryptamine	Alkaloid	5-HT receptor modulator	[247]
Barettin	Brominated cyclodipeptide	5-HT receptor modulator	[248]
Damipipecolin	Bromopyrrole alkaloid	5-HT receptor modulator	[249]
Damituricin	Bromopyrrole alkaloid	5-HT receptor modulator	[249]
Aplysamine-1 and analogs	Bromotyrosine derived metabolite	histamine receptor modulator	[160,250]
Aplysinopsin and analogs	Indole alkaloid	Glycine receptor modulator	[251]
χ-MrIA	χ-Conotoxin	Norepinephrine transporter modulator	[166]
Astaxanthin	Carotenoid	Neuroprotection against 6-OHDA or MPP^+^ treatments	[252]
Nanolobatolide	C_18_-terpene	Anti-neuroinflammatory activity	[253]
Δ9(12)-Capnellene-8β,10α-diol	Sesquiterpene	Anti-neuroinflammatory activity	[254,255]
(−)-8,15-Diisocyano-11(20)-amphilectene	Diterpene isocyanide	Anti-neuroinflammatory activity	[256]
Monamphilectine A	Diterpene isocyanide	Anti-neuroinflammatory activity	[257]
Pseudopterosin	Diterpene	Anti-neuroinflammatory activity	[258,259,260,261]
Eusynstyelamide A	Brominated tryptophan-derivative	Anti-neuroinflammatory activity	[262]

## 11. Conclusions

Complete recovery from a CNS injury or disorder is not yet a reality. Regeneration of parts of the brain, where loss of large amount of neurons occurred, is very difficult. Drugs only alleviate the symptoms and/or delay the progression of the injury or disease, and cell and tissue implantation are still in their infancy. Therefore, the search for new neuroprotective drugs is still an urgent matter, and natural products isolated from marine invertebrates are excellent candidates for drug development programs. This review intended to update the state of the art on this subject and to show how marine invertebrates neuroactive drugs affect neuronal growth and synaptic functions, neurodegeneration and neuroinflammation. We should continue to be optimistic about the future of therapy development for CNS disorders and continue to explore the marine environment, which is an inexhaustible source of neuroactive drugs.
